# Microglia aggregates define distinct immune and neurodegenerative niches in Alzheimer's disease hippocampus

**DOI:** 10.1007/s00401-025-02857-8

**Published:** 2025-02-15

**Authors:** Sonja Fixemer, Mónica Miranda de la Maza, Gaël Paul Hammer, Félicia Jeannelle, Sophie Schreiner, Jean-Jacques Gérardy, Susana Boluda, Dominique Mirault, Naguib Mechawar, Michel Mittelbronn, David S. Bouvier

**Affiliations:** 1 Luxembourg Centre for Systems Biomedicine (LCSB), University of Luxembourg, Belval, Luxembourg; 2 Luxembourg Center of Neuropathology (LCNP), Dudelange, Luxembourg; 3 Laboratoire National de Santé (LNS), National Center of Pathology (NCP), 1, Rue Louis Rech, 3555 Dudelange, Luxembourg; 4 Department of Cancer Research (DOCR), Luxembourg Institute of Health (LIH), Strassen, Luxembourg; 5 Department of Neuropathology, Pitié-Salpêtrière Hospital, AP-HP Sorbonne University, Paris, France; 6 Institut du Cerveau, Paris Brain Institute, ICM, Inserm U1127, CNRS UMR7225, APHP, Sorbonne University, Pitié-Salpêtrière Hospital, Paris, France; 7 Douglas Mental Health University Institute, McGill University, Montreal, QC Canada; 8 Department of Psychiatry, McGill University, Montreal, Quebec Canada; 9 Department of Life Sciences and Medicine (DLSM), University of Luxembourg, Esch-sur-Alzette, Luxembourg; 10 Faculty of Science, Technology and Medicine (FSTM), University of Luxembourg, Esch-sur-Alzette, Luxembourg

**Keywords:** Spatial profiling, Alzheimer's disease, Hippocampus, Glial cells, Proteinopathies, Peripheral immune cells

## Abstract

In Alzheimer’s disease (AD), microglia form distinct cellular aggregates that play critical roles in disease progression, including Aβ plaque-associated microglia (PaM) and the newly identified coffin-like microglia (CoM). PaM are closely associated with amyloid-β (Aβ) plaques, while CoM are enriched in the pyramidal layer of the CA2/CA1 hippocampal subfields, where they frequently engulf neurons and associate with tau-positive tangles and phosphorylated α-synuclein. To elucidate the role of these microglial subtypes, we employed high-content neuropathology, integrating Deep Spatial Profiling (DSP), multiplex chromogenic immunohistochemistry and confocal microscopy, to comprehensively map and characterise their morphological and molecular signatures, as well as their neuropathological and astrocytic microenvironments, in AD and control post-mortem samples. PaM and PaM-associated astrocytes exhibited signatures related to complement system pathways, ErbB signalling, and metabolic and neurodegenerative processes. In contrast, CoM displayed markers associated with protein degradation and immune signalling pathways, including STING, TGF-β, and NF-κB. While no direct association between CD8 + T cells and either microglial type was observed, CD163 + perivascular macrophages were frequently incorporated into PaM. These findings provide novel insights into the heterogeneity of microglial responses, in particular their distinct interactions with astrocytes and infiltrating immune cells, and shed light on specific neurodegenerative hotspots and their implications for hippocampal deterioration in AD.

**Supplementary Information:**

The online version contains supplementary material available at 10.1007/s00401-025-02857-8.

## Introduction

Alzheimer's disease (AD) is a multifaceted disease characterised by numerous molecular and cellular alterations that remain incompletely understood. Recognised as the most common age-related neurodegenerative disease (NDD) and the leading cause of dementia, AD is characterised by a progressive and irreversible decline in cognitive function, with memory loss as one of the most prominent clinical symptoms. The cellular underpinnings of memory loss are linked to an increased vulnerability of anatomical brain regions associated with memory, such as the hippocampus, to AD [1, 4, 39, 45, 50, 67, 75, 110]. Memory-related brain regions are particularly affected by atrophy, neuronal and synaptic loss, neuroinflammation and high burdens of extracellular amyloid-β (Aβ) plaques and intraneuronal hyperphosphorylated tau (pTau) neurofibrillary tangles (NFTs), the latter two being the neuropathological hallmarks of AD. Co-occurrence with other misfolded protein pathologies, such as phosphorylated α-synuclein (pSyn) typical of Parkinson’s disease (PD), or TAR DNA-binding protein 43 (TDP-43), found in pathological deposit in amyotrophic lateral sclerosis (ALS) or frontotemporal dementia (FTD) is frequently observed [29, 36, 54, 55, 57, 69, 73].

While traditionally considered secondary players in neurodegenerative processes, the roles of glial cells in AD progression have received increasing attention. Microglia, the immune cells of the central nervous system (CNS), are critical for maintaining brain homeostasis [26, 78, 84]. In AD, they may be directly involved in neurodegeneration. Genome-wide association studies (GWAS) identified AD risk factors associated with microglial function such as TREM2, ApoE or CD33, highlighting their importance in disease pathogenesis [8, 35, 44, 63, 85, 92, 107]. Single-cell profiling have revealed subpopulations of microglia which could be directly involved in AD progression [25, 28, 40, 43, 52, 53, 93]. We have recently shown that some morphologically defined microglial clusters are associated with specific protein pathologies and display sub-regional heterogeneity in the hippocampus of AD and dementia with Lewy Bodies (DLB) patients [22], with the most pronounced changes being seen in the cornu ammonis (CA) 1 of AD patients. Indeed, the ability of microglia to regroup into cellular aggregates, or clumps, around an insult is a common indicator of degenerative hotspots. Microglial accumulation in a rosette-like conformation around Aβ core plaques in post-mortem human AD samples or mouse models [3, 11, 19] has been particularly studied [43, 104, 109]. Yet the function of plaque-associated microglia (PaM) remains controversial, with evidence suggesting both protective and pathological roles to AD. PaM are associated with Aβ core plaque clearance [30] and formation [32, 86, 98], with lipid metabolism [31, 83] and local neuroinflammation [34, 47, 101]. Additionally, various types of microglial accumulations have been observed in numerous brain diseases such as microglia nodules or white matter-associated microglia (WAMs) in mouse models of inherited ALS [21, 94], AD [76], human multiple sclerosis (MS) [13] or viral encephalitis [95] and COVID-19 [80] brains, highlighting the complexity of microglial involvement in neurodegenerative processes. In addition to microglia, astrocytes undergo morphological and molecular changes in AD, reflecting their numerous contribution to CNS homeostasis, defence and immunity [10]. While less characterised than microglia, astrocytes may have a significant impact on the progression of AD by shifting some protective phenotypes to detrimental ones. Polarised and reactive astrocytes are found enclosing PaM, to form together an organised structure, the so-called reactive glial net (RGN) [11], and may regulate the Aβ plaque cellular surrounding architecture and spacing [33]. Moreover, infiltrating peripheral immune cells such as T cells or macrophages, which could drastically impact inflammation and microglial responses, have been described to reach Aβ plaques in AD mouse models and human post-mortem samples [91]. However, the roles of microglia, astrocytes and infiltrating immune cells in the rapid degeneration of the hippocampus still need to be further characterised.

Here we have found two morphologically distinct types of microglial accumulations in the hippocampus of late-onset AD (LOAD) patients, the typical PaM surrounding Aβ plaques, and a newly defined accumulation termed coffin-like microglia (CoM) that was specifically enriched in the CA1 pyramidal layer (PL), but much less abundant than PaM. We aimed to elucidate molecular and morphological characteristics of CA1 PaM and CoM, surrounding astrocytes and their association with infiltrating immune cells using a cohort of 52 AD, DLB and control (CTL) human post-mortem samples. To this purpose, we employed spatial protein and transcriptome profiling [51, 88], high- and super-resolution confocal microscopy, chromogenic multiplexing immunohistochemistry and digital pathology.

Our analysis unveiled a comprehensive new set of features for microglia and astrocytes in AD that are specific to their neurodegenerative microenvironment and show an association with infiltrating immune cells at the level of Aβ plaques. Our study advances the characterisation of glial-immune hotspots in neurodegeneration of the hippocampus and beyond. Furthermore, our findings have the potential to inform the development of novel therapeutic strategies targeting glial and immune cells to mitigate neurodegenerative processes.

## Materials and methods

### Human brain samples

Human pseudonymized paraformaldehyde (PFA)-fixed and formalin fixed paraffin embedded (FFPE) post-mortem mid-hippocampal (between the uncus and *corpus geniculatum laterale*) samples were obtained from the Douglas-Bell Canada Brain Bank (Douglas Mental Health University Institute, Montreal, QC, Canada) and GIE-Neuro-CEB biobank (Groupe Hospitalier Pitié-Salpêtrière, Paris, France). Their use for research was approved by the respective Ethic Panels of the aforementioned, as well as of the University of Luxembourg (ERP 16–037 and 21–009).

The neuropathological diagnosis was performed by brain bank intern neuropathologists following Braak [12], ABC staging [60] and McKeith staging criteria [56] based on Aβ plaques, NFTs and α-synuclein.

PFA-fixed hippocampal samples include non-demented age-matched controls (CTLs, *n* = 12), DLB patients (*n* = 4) and AD patients (*n* = 23). FFPE hippocampal and cortical samples include CTLs (*n* = 8) and AD patients (*n* = 19). 3 of the CTLs and 11 of the AD cases were available both as PFA-fixed and FFPE material. Age at death, post-mortem delay (PMD), sex and disease score of the human subjects are listed in Supplementary Table 1.

### Immunohistochemistry (IHC)

FFPE hippocampal blocs were cut using a standard microtome into 5 µm-thick sections, mounted on coated Dako FLEX IHC microscope slides (Cat# K8020, Agilent). The slides were dried in an oven at 60 °C for at least 60 min. All the slides were processed on an automated immunostainer (Dako Omnis Immunostainer, Agilent) with heat induced epitope retrieval (HIER) (EnVision^TM^FLEX Target Retrieval Solution high or low pH buffer, Cat# K8004 and Cat# K8005, respectively) for 30 min at 97 °C. Primary antibodies (anti-Iba1 Cat# 019–19741, anti-cd68 Cat# 916,104, anti-STING Cat# MA5-26,030, anti-C1q Cat# ab182451, anti-ITGA6 Cat# HPA012696, anti-ACADS Cat# HPA022271; antibody concentrations in Supplementary Table 2) were diluted in EnVision^TM^FLEX antibody diluent (Cat# K8006). Primary antibody incubation lasted for 1h at room temperature (RT), detection based on horseradish peroxidase (HRP)/ 3, 3′-diaminobenzidine (DAB) substrate system (EnVision^TM^FLEX Detection kit, Cat# K8000) and, finally, counterstained with haematoxylin (Cat# GC808). Finally, the slides were dehydrated through ethanol baths and mounted.

### Multiplex chromogenic IHC

For all experiments made using Ventana Discovery Ultra automated IHC/in situ hybridization (ISH) research platform (Roche Ventana Medical Systems, Tucson, AZ, USA), 5 µm sections from FFPE blocks were mounted on Matsunami TOMO® hydrophilic adhesion glass slides (Cat# TOM-1190). Slides were dried as previously described. All reagents were included in the system as recommended by Roche Diagnostics and washing was performed in between steps with Reaction buffer (Cat# 950–300). All antibody concentrations are listed in Supplementary Table 2. For single and multiplex IHC, counterstain was done with 4 min each incubation of haematoxylin II (Cat# 790–2208) and bluing reagent (Cat# 760–2039). Consecutively, all slides were washed, dehydrated through a series of alcohols and coverslipped.

For DAB single-plex IHC, sections were first deparaffinized at 69 °C in EZ Prep solution (Cat# 950–102) for 8 min during 3 cycles. HIER was performed at 95 °C using Cell Conditioning 1 (standard CC1, Cat# 950–224) reagent for 40 min. For blocking endogenous peroxidases and proteins, slides were incubated with inhibitor ChromoMap (CM) at 37 °C for 8 min. Then, primary antibodies (anti-C3 Cat# HPA003563, anti-C4d Cat# ab36075, anti-PCSK9 Cat# MA5-32,843) were added by manual application (diluted in EnVision^TM^FLEX). After 60 min incubation with primary antibodies, OmniMap anti-Rb (Cat# 760–4311) or OmniMap anti-Ms HRP (Cat# 760–4310) was applied and incubated for 16 min, followed by 4 min of H_2_O_2_ CM, 8 min of DAB CM and 4 min of Copper CM from the Discovery CM DAB kit (Cat# 760–159).

All the multiplex protocols (two- to four-plex) followed the same initial steps of deparaffinization, HIER with CC1, and blocking with inhibitor CM, unless stated otherwise.

In the two-plex CD163/Iba1, after deparaffinization and HIER steps, one drop of Discovery inhibitor (Cat# 760–4840) was applied for 8 min. Then, anti-CD163 (Cat# 760–4437) was incubated for 60 min and developed by OmniMap anti-Ms HRP during 16 min, followed by 4 min of Discovery Purple and 32 min of H_2_O_2_ Purple from the Discovery Purple Kit (Cat# 760–229). Heat mediated (100 °C, 24 min) antibody denaturation was performed using Cell Conditioning 2 (CC2) reagent (Cat# 950–223) prior to each cycle of sequential staining in every panel to denature the primary antibody-HRP complex and prevent subsequent chromophores of binding to any residual unbound HRP-conjugated primary antibody. Then, anti-Iba1 (Cat# 019–19741) was added for 48 min. The signal was amplified with OmniMap anti-Rb HRP for 16 min followed by a Discovery Teal HRP chromophore (Cat# 760–247) consisting of an incubation of 4 min of Teal HRP Substrate, 32 min of Teal HRP H_2_O_2_ and 16 min of Teal HRP Activator.

The three-plex experiments were conducted as follows: for the CD163/Iba1/4G8 combination, anti-CD163 was incubated as before and stained with Discovery CM DAB as described in single-plex protocol. After denaturation, anti-Iba1 was applied during 48 min, followed by OmniMap anti-Rb HRP for 16 min and then Discovery Purple Kit. For the last step, anti-4G8 (Cat# 800-712) was incubated 1 h and amplified with OmniMap anti-Ms HRP for 16 min, followed by a Discovery Teal HRP.

For the Iba1/4G8/GFAP and PS396/4G8/GFAP combinations, anti-Iba1 and anti-PS396 (Cat# 44-752G) were incubated, respectively, 1 h 32 min and 1 h, followed by OmniMap anti-Rb HRP incubation (20 min) and DAB staining as in the single-plex IHC. Subsequently, anti-4G8 was added for 60 min followed by 12 min incubation with UltraMap anti-mouse Alk Phos (AP) (Cat# 760–4312) for 12 min. Then DISCOVERY Yellow kit (RUO, Cat# 760–239) consisting of 4 min of Yellow buffer and 44 min of Disco Yellow was applied. Finally, anti-GFAP (Cat# 760–4345) was applied for 40 min, followed by OmniMap anti-Rb HRP for 16 min and Discovery Teal HRP.

The four-plex IHC, Iba1/AT8/4G8/pSer396 and Iba1/CD3/4G8/CD8 followed a similar protocol. Briefly, anti-Iba1 was applied for 1 h 32 min followed by OmniMap anti-Rb HRP incubation (20 min) and Discovery CM DAB kit. Anti-AT8 (Cat# MN1020) and anti-CD3 (Cat# 790–4341) were incubated, respectively, for 60 or 32 min, followed by, respectively, OmniMap anti-Ms HRP or OmniMap anti-Rb HRP for 16 min and coupled with Discovery Purple kit. Then, anti-4G8 yellow staining was done as in the previous three-plex. Finally, anti-pSer396 (Cat# 44-752G) or anti-CD8 (Cat# 790–4460) was added for, respectively, 60 or 40 min, amplified with OmniMap anti-Rb HRP for 16 min and coupled to the Discovery Teal HRP.

### Fluorescent IHC

PFA-fixed samples were cut and immunostained as previously described [22, 68, 77]. Briefly, PFA-fixed samples were washed in phosphate-buffered saline (PBS), cryo-preserved in 30% sucrose and cut into 80 µm thick sections on a sliding freezing microtome (LeicaSM2010R). After overnight ultraviolet (UV) irradiation for autofluorescence reduction the sections were permeabilized through 30 min incubation with 0.5% Triton-X 100 prior the blocking step with 2% horse serum for 2 h. The sections were incubated with primary antibodies in blocking solution with concentrations in Supplementary Table 2 (anti-Aβ Cat# 800,712, anti-GFAP Cat# 173,004, anti-Iba1 Cat# 019–19741, anti-Iba1 Cat# LS-B2402, anti-pSyn Cat# NA, anti-pTau Cat# MN1020, anti- NF-κB Cat# ab86299, anti-pMLKL Cat# OASG07777, anti-erbB4 Cat# HPA012016, anti-C3 Cat# HPA003563, anti-ITGA6 Cat# HPA012696, anti-SMURF2 Cat# HPA071508, anti-ACADS Cat# HPA022271), for 72 h at 4°C. Afterwards slices were washed with PBS and incubated with secondary antibodies (Donkey anti-mouse Alexa Fluor 488 Cat# 715–545-150, Donkey anti-goat Alexa Fluor 488 Cat# 705–545-147, Donkey anti-guinea pig Alexa Fluor 488 Cat# 706–545-148, Donkey anti-rabbit Alexa Fluor 555 Cat# A-31572, Donkey anti-guinea pig Alexa Fluor 647 Cat# 706–605-148, Donkey anti-mouse Alexa Fluor 647 Cat# 715–605-150, Donkey anti-goat Alexa Fluor 647 Cat# 705–605-003, Goat anti-rabbit Atto 647N Cat# 40,839) for 2 h at RT. After 3 washing steps with PBS, some sections were counterstained with DRAQ7™ far-red fluorescent DNA dye (Cat# 7406, 20 min; RT) to label nuclei or with 0.2 μM Thiazine red (TR) solution (Sigma Chemicals, St. Louis, MO, USA) for 20 min at RT to stain Aβ plaques and tau fibrils. Samples were then washed two times for 10 min in PB prior to mounting on glass slides (Superfrost Microscope slides) using ProLong Gold Antifade reagent (Cat# P36930).

### Bright-field microscopy

Bright-field images of chromogenic stained FFPE sections were acquired at 5,10, 20 and 40X objectives of a Leica bright-field DM2000 LED microscope and a Leica DMC2900 camera (Leica Microsystems) or by a high-throughput bright-field slide scanner (IntelliSite Ultra Fast Scanner, Philips).

### Confocal and stimulated emission depletion (STED) microscopy

Three dimension (3D) z-stack confocal acquisitions were obtained by LSM710 and LSM800 confocal microscopes with 20X air (Zeiss Plan-APOCHROMAT 20x/0.8 420,650–9902) and 40X oil (Zeiss EC Plan-NEOFLUAR 40x/1.3 Oil DIC 420462–9900) objectives.

3D STED z-stack acquisitions were obtained with a Leica DMi8 with TCS SP8 microscope equipped with 100X oil STED objective (Leica HC PL APO 100x/1.40 OIL STED white ∞0.17/OFN25/D) and 775nm STED depletion laser. STED images were deconvolved with Huygens Professional software (Scientific Volume Imaging). Tile scans were stitched using the LAS X software (Leica Microsystems), Zeiss microscope software ZEN or Imaris Stitcher (Supplementary Table 3). 3D stacks were visualised with Imaris (9.6.0 and 9.8.0) software after each channel underwent intensity normalisation and background subtraction to remove noise.

### Digital pathology

All chromogenic IHC image analyses were performed using HALO® image analysis platform (Indica Labs, version 3.6). Hippocampi were manually divided into 4 sub-regions (CA4, dentate gyrus (DG), CA3 and CA2/CA1) and their areas were measured. CD68 quantification was performed using the HALO® area quantification module, which measures stain-positive areas. CD8 + Tcells were quantified manually and differentiated between cells found in blood vessels (BV) and parenchyma. Percentages of stained area or cell count (cells/mm^2^) were compared using *t *tests with adjustment for multiple comparisons (using Holm’s method). To quantify the number of PaM and PaM astrocytes, but also CD163 + and CD163 + Iba1 + double-positive cells associated with Aβ plaques and assess their relationship to the area of the plaques in the CA1 subfield of the hippocampus, we first manually delineated the surface of each plaque and then, manually counted the number and type of cells attached (color based). We then compared the mean number of cells attached to 1000 µm^2^ of amyloid area using *t* tests with adjustment for multiple comparisons (Holm’s method). Then we described the Pearson correlation between cell type and plaque area using ordinary least squares linear regression.

### Volumetric quantification and nuclei count

Volume and nuclei count of 61 microglia accumulations were assessed in high-resolution confocal acquisitions (40x) of Iba1 and DRAQ7 ™ immunostained hippocampal sections from age-matched CTLs (*n* = 1), AD (*n* = 8) and DLB (*n* = 4) subjects. Using the surface tool from the Imaris (9.8.0) software, microglia accumulations were 3D reconstructed (surface detail parameter of 400µm) and volume was extracted. Subsequently, nuclei of each accumulation were manually marked and counted with the spots tool.

### Deep spatial profiling (DSP)

#### Sample preparation for DSP

Postmortem FFPE hippocampus blocs from three AD patients (cases #49, #50 and #52; Supplementary Table 1) were cut into 5 µm-thick sections on a microtome. Sections were mounted on microscopy slides, without coverslip (Thermo Scientific Superfrost Plus, J1800AMNZ), to produce three slides with each containing one section of all AD patients (three sections per slide) that were cut at different depths to avoid overlap of identical microglia accumulations. Finally, slides were dried overnight at 37 °C in an oven. Two slides were used for spatial transcriptomic analysis and one slide for spatial proteomic analysis.

#### Spatial proteomics (SP)

GeoMx DSP technology (NanoString Technologies) was used to perform cell-type enriched spatial proteomic analysis. Areas of illumination (AOIs) were manually drawn on the GeoMx DSP analysis suite (Version 2.5.1.145) software based on fluorescent antibody morphology markers including anti-Iba1 (Cat# 48-934), anti-GFAP (Cat# NBP2-33184DL594), anti-Aβ (Cat# NBP2-13075AF532) and SYTO™ 13 green fluorescent nucleic acid stain (Cat# S7575). PaM and CoM were distinguished through, respectively, presence or absence of Aβ plaques and Iba1-positive area, as well as surrounding areas were segmented. In total 24 AOIs for segmentation were selected: 7 PaM-Iba1, 7 PaM-Surrounding, 5 CoM-Iba1 and 5 CoM-Surrounding. The Human Protein Next Generation Sequencing (NGS) panel probe mix was used and contained in total 147 targets from the following modules: human protein core for NGS, immune cell typing, immune activation status, 10 drug target, pan-tumour, cell death, MAPK signalling, P13K/AKT signalling, myeloid, autophagy, neural cell typing, AD pathology, AD pathology extended, PD pathology and glial cell subtyping.

Upon AOI specific UV illumination, indexing oligonucleotide tags from the probe mix were released, collected in a 96-microwell plate and analysed on an nCounter® platform. FASTQ files for Nanostring GeoMx were aggregated into count matrices based on Unique Molecular Identifier (UMI) and molecular target tag sequences. Single probe genes were reported as the deduplicated count value. The digital counts were normalised to negative probes (Ms IgG1, Rb IgG, Ms IgG2a).

#### Spatial transcriptomics (ST)

GeoMx DSP technology (NanoString Technologies) was used to perform cell-type enriched spatial transcriptomic analysis. AOIs were selected based on the identical morphology markers as for the spatial proteomic analysis. AOIs from three AD patients (in duplicate but at different depths levels) were localised in the pyramidal layer of the hippocampus. PaM and CoM AOIs were identified as previously described and Iba1-positive areas were segmented as well as GFAP-positive area. For rod-shaped microglia (Rod) AOIs exclusively Iba1-positive area was segmented. In total 38 AOIs for segmentation were selected: 10 PaM-Iba1 AOIs, 10 PaM-GFAP AOIs, 7 CoM-Iba1, 7 CoM-GFAP and 4 ROD-Iba1 AOIs. The probes in overlapping areas of Iba1 and GFAP staining were included in Iba1 segmentation. The probe mix of 18,677 ISH probes conjugated to UV-photocleavable indexing oligonucleotide tags (Transcriptomics Probekit (v1.0) Human NGS Whole Transcriptome Atlas RNA) were added to the slide and indexing oligo sequences were released and collected in all AOIs and counted.

We removed targets with expression lower than 5% segments above limit of quantification (LOQ). We then scaled the dataset to the geometric mean of nuclei and finally performed quartile (Q) 3 normalisation, creating a dataset with 15,518 transcripts.

#### Over-representation analysis (ORA)

We performed ORA using the R package clusterProfiler [108] for differentially expressed genes (DEGs) having a log-fold change lower than – 0.5 or greater than 0.5 and associated *P* value < 0.1 after curating the database for all microglia-associated or astrocytes-associated transcripts validated in the literature and in online databases such as BrainRNAseq (https://brainrnaseq.org) or astrocyteRNAseq (http://astrocyternaseq.org) for astrocytes. We employed the following databases: Gene Ontology (GO), Kyoto Encyclopedia of Genes and Genomes (KEGG) and Wikipathways (WP).

#### Quantification and statistical analysis

For the volume quantification and nuclei count analysis (61 microglia accumulations) a Kruskal–Wallis test by ranks was performed using GraphPad Prism software (Version 9.4.1). In the spatial proteomic analyses of 147 target proteins in 24 AOIs differentially expressed proteins (DEPs) were detected by applying two-sided unpaired *t* tests. In the spatial transcriptomic analyses of target transcripts in 38 AOIs, DEGs were detected by applying unpaired *t* tests assuming a normal distribution of the data. All *p* values were adjusted for multiple comparisons using the Benjamini–Hochberg procedure with false discovery rate (FDR) of 0.1. Heatmaps and volcano plots were generated in R (version 4.2.1.) using the *pheatmap* (version 1.0.12) and *ggplot* (version 3.3.6) packages. In this manuscript and supplementary files, * indicates *P* < 0.05, ** *P* < 0.01 and *** *P* < 0.001. In Supplementary Excel 1 for multiple groups of comparisons, p.adj denotes *p* values adjusted by the Benjamini–Hochberg procedure for one single group of comparisons, while p.adj2 is adjusted for the entirety of comparisons.

## Results

### Microglia regroup into morphologically distinct cell aggregates in AD hippocampi

**Fig. 1:**
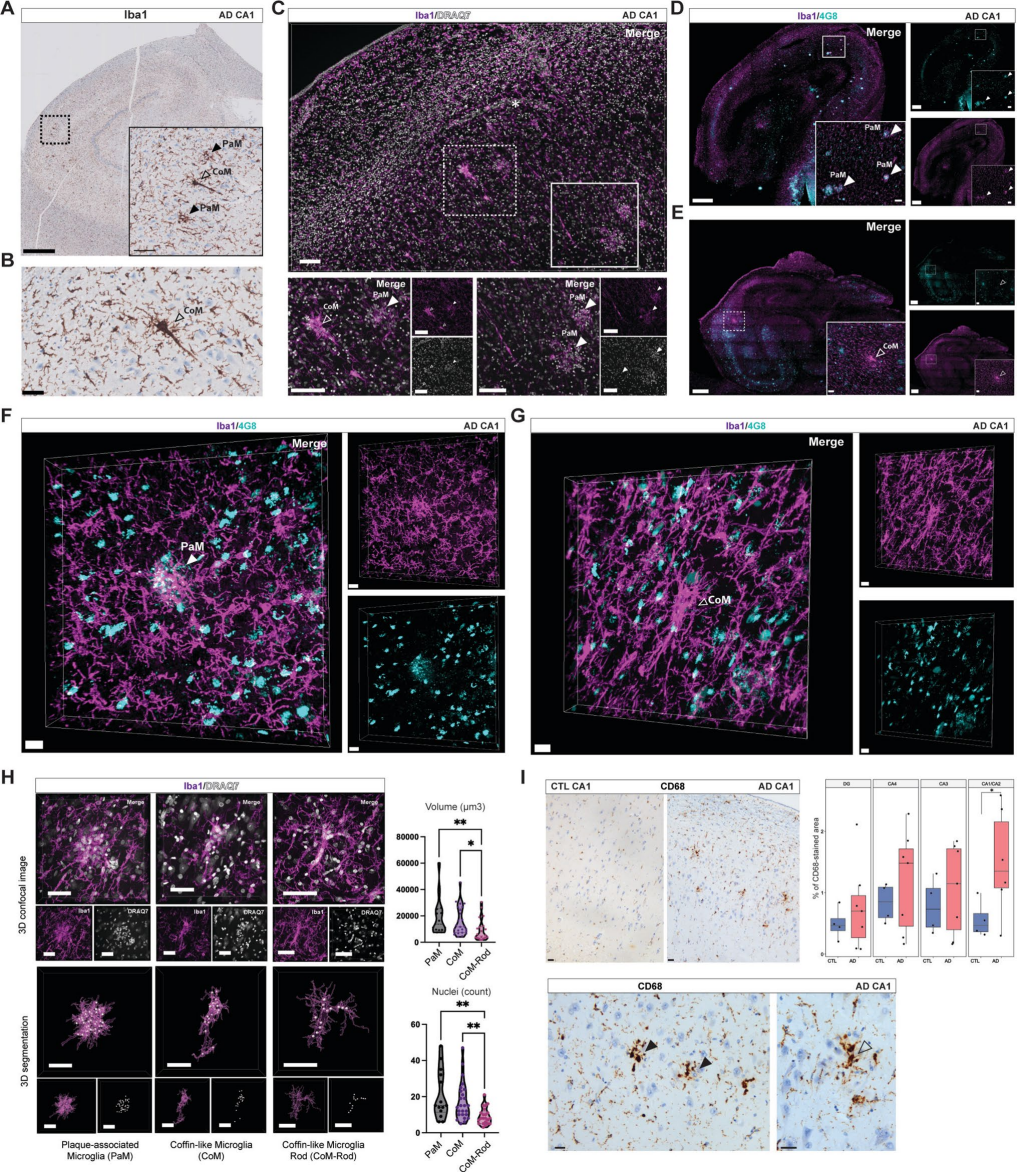
PaM and CoM have a distinct morphology and relationship to Aβ plaques in hippocampi of AD patients. **A**–**C** Representative images of PaM and CoM in human post-mortem hippocampal AD tissue (case #52). **A**, **B** Consecutive FFPE sections stained with Iba1 (brown) and nuclei (haematoxylin, blue). **C** PFA-fixed section stained against Iba1 (magenta) and nuclei (DRAQ7™, white). Both staining methods reveal typical rosette distribution of PaM (full arrows) and polarised accumulations of microglia termed CoM (empty arrows) in the CA1 PL. The asterisk indicates a blood vessel to highlight that these accumulations are not associated with the vasculature. **D**, **E** Confocal overview of Iba1 (magenta) and 4G8 (teal) stainings in AD hippocampi showing the co-localisation between PaM (full arrows) and Aβ plaques (**D**, case #35) while CoM are spatially distant from Aβ plaques (empty triangles) (**E**, case #38). (**F**, **G**) High-resolution 3D confocal images showing the morphological differences between PaM (**F**, case #25) and CoM (**G**, case #35) and their respective interaction with Aβ plaques. **H** Quantification of average volume and number of nuclei of PaM, CoM and CoM-Rod across our collection of AD and DLB and age-matched CTL samples. Images showing confocal 3D stacks of microglia (Iba1, magenta) and nuclei (DRAQ7™, white) with corresponding 3D segmentations with violin plot of volume (µm^3^) and nuclei (count) of PaM, CoM and CoM-Rod. **I** CD68 (brown) expression is increased in AD CA1 (case #52) compared to CTL (case #7) and found in PaM (full arrows) and CoM-like accumulations (empty arrows). Scale bars: **A** 1mm (low mag), 100µm (high mag); **B**, **C** 100 µm **D**, **E** 1 mm (low mag), 100 µm (high mag); **F**, **G** 20 µm; **H** 50 µm; **I** 30 µm

To elucidate the alterations of microglia within the hippocampus of AD patients, we performed IHC analysis of the microglial morphological marker, ionised calcium-binding adaptor molecule 1 (Iba1), in a cohort of CTLs and AD post-mortem FFPE samples. We identified two distinct types of large Iba1-positive cellular accumulations. The most common were the typical Aβ plaque-associated microglia (PaM), characterised by a high number of microglia clustered around an Aβ core plaque in a rosette-like conformation. Less frequently encountered, but consistently present in our sample collection, were clusters of microglia with a polarised shape reminiscent of a 'soma' and 'tail' configuration, observed predominantly in the PL of the CA1 and CA2 regions of the hippocampus (Fig. 1A, B). Across serial sections of hippocampi from 16 AD cases and 5 age-matched CTLs, we observed that this type of accumulation occurred in the CA region of AD patients at a ratio of approximately 1 to 11 compared to PaM. This accumulation was absent in CTL samples and was not detected in prefrontal, temporal, or entorhinal cortices of either CTL or AD cases.

To further characterise the 3D morphological features and differences from PaM, we immunostained PFA-fixed 80 µm hippocampal sections from AD patients for Iba1 and 4G8 (Aβ). Using confocal and STED microscopy, we confirmed the enrichment of this peculiar type of Iba1- accumulation in the CA1 and CA2 PL of AD hippocampi (Fig. 1C) and showed that they were spatially separated from 4G8-positive Aβ plaques (Fig. 1D–G, Supplementary Fig. 1A, B). High and super-resolution microscopy revealed new features of their complexity with Iba1-cells showing tight boundaries and an encapsulating cellular organisation that was clearly distinct from PaM [11, 19] with a size and consistent orientation resembling that of pyramidal neurons (Supplementary Fig. 1, Supplementary Movie 1; Supplementary Movie 2). Co-staining with DRAQ7™ often showed nuclei that appeared to be engulfed by Iba1-positive cells (Supplementary Movie 1, Supplementary Fig. 2). These microglia collectively formed a tight shell around as yet-unidentified structures, resembling the glial embrace (“Gliöse Umklammerung”) or coffin formation (“Totenladenbildung”) around neurons observed in various conditions in the early twentieth century literature [2, 27, 41, 87, 89], that we hereafter referred to as “coffin-like microglia” or CoM (Fig. 1A, C, E, G; Supplementary Fig. 1B, D).

Our high-resolution 3D analysis allowed us to distinguish between two types of CoM. We noticed some CoM formations were predominantly composed of bipolar or rod-shaped microglia, resulting in a thin and elongated structure in 3D, often traversing the PL, while only partially enveloping surrounding cells. Unlike the first CoM described, the other type did not seem to enclose any other structure. We termed them CoM-Rod (Fig. 1H, Supplementary Fig. 3). In addition, we often observed smaller groups of Iba1-positive cells 'clumped' together, located between other brain cells (Supplementary Fig. 3). Our previous findings indicated that morphological alterations of individual microglia in DLB resembled to those observed in AD, but with less severity [22]. Consistent with these observations, we detected PaM, CoM, CoM-Rod, and clumped microglia to a lesser extent in the hippocampi of DLB patients (Supplementary Fig. 1C, D).

We quantified CA1/CA2 PaM and CoM number of cells and their total volume from 3D confocal stacks (40x). We did not observe CoM in age-matched CTL samples (*n* = 8), however, we found few PaM and CoM-Rod. Using Imaris software, we quantified volumes and number of nuclei contained in 61 3D z-stack images of PaM, CoM and CoM-Rod across age-matched control (*n* = 1), AD (*n* = 8) and DLB patients (*n* = 4) samples (Fig. 1H). PaM presented a mean volume of 19,744 µm^3^ with an average of 20 nuclei, CoM had a mean volume of 15,776 µm^3^ and an average of 17 nuclei, and CoM-Rod a mean volume of 8,870µm^3^ and an average of 9 nuclei (Fig. 1H). Using the Kruskal–Wallis test by ranks, we showed that CoM-Rod are significantly smaller in volume (*P* < 0.05, *P* < 0.01 respectively) and in nuclei count (*P* < 0.01) than CoM and PaM. Even though PaM and CoM are very different in morphology, their volume and nuclei count did not present significant differences.

We then investigated the association of a typical marker of microglia upregulated in disease, cluster of differentiation 68 (CD68) [9, 30, 71]. We first quantified CD68 expression in hippocampal subfields of CTL and AD FFPE samples (*n* = 7 CTL, 8 AD). CD68 was expressed in microglia-like cells in CTL and AD in all brain regions examined, slightly increased in all subfields but more drastically in the CA1 AD (*P* < 0.05). We found CD68 associated with PaM and CoM-like structures in the CA hippocampus, showing a shared feature of PaM and CoM (Fig. 1I).

### PaM and CoM exhibit distinct pathological and astrocytic microenvironments

**Fig. 2:**
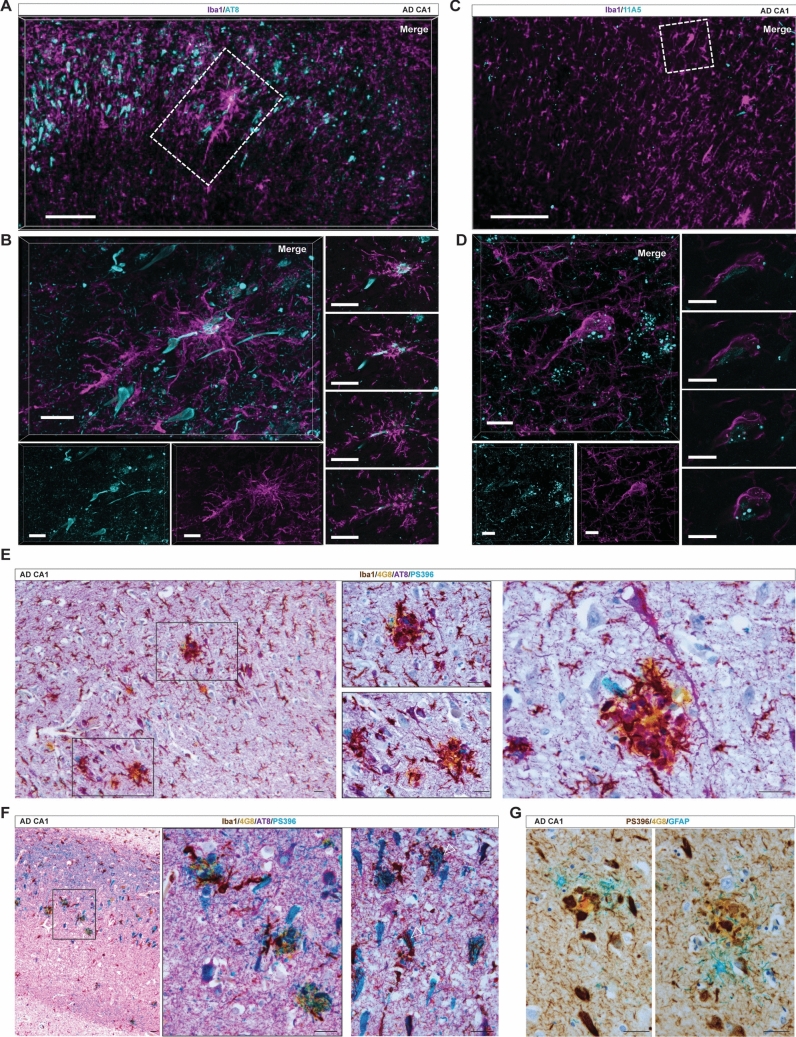
CoM and PaM are both associated with tau pathology. **A**, **B** CoM (Iba1, magenta) are associated with AT8 positive NFTs (cyan) in the PL of CA1 in AD patients (case #23). High-resolution of the CoM reveals its polarised architecture in 3D (left panels) and enwrapping along the NFT in 2D z-stacks (right panels). **C**, **D** CoM (Iba1, magenta) can encompass neuronal cells carrying intraneuronal granular pSyn inclusions (11A5, cyan) in the hippocampus of AD patients (AD, case #35). High-resolution of the CoM shows its polarised architecture in 3D (left panels) and close aggregation around the cell bearing granular pSyn inclusion in 2D z-stacks (right panels). **E**, **F** 4-plex chromogenic IHC for Iba1 (DAB, brown), AT8 (purple), 4G8 (yellow) and PS396 (teal) highlighted the accumulation of swollen AT8 + PS396 + (dark blue) structures near plaques. We found neurons with tangles that were AT8 + only, PS396 + only or double positive. AT8 + PS396 + were often surrounded by microglia, sometimes forming aggregates reminiscent of CoM (empty arrows) (cases #30, #48 and #49). **G** When GFAP staining (teal) is combined with that of 4G8 (yellow) and PS396 (DAB, brown), it reveals the encapsulation of plaque-associated PS396 by PaM astrocytes (case #48). Scale bars: **A** 200 µm; **B** 50 µm; **C** 100 µm; **D** 20 µm; **E**–**G** 30 µm

To elucidate the relationship of PaM and CoM with surrounding cells, tau and α-synuclein pathologies, we investigated the characteristics of their pathological microenvironment. Given the distinct association of PaM and CoM with Aβ plaques, we further investigated their interactions with pTau NFTs and pSyn aggregates in AD [22, 97]. Using high-resolution confocal imaging of immunostained sections for Iba1 and AT8 (pTau), we found that CoM can fully encompass NFT-bearing neurons, enveloping the entire 'flame' shape of the tangle and spreading to surrounding neuronal cells (Fig. 2A, B, Supplementary Movie 3). Using thiazine red (TR), which binds β-pleated sheet structures, we also observed CoM containing TR-positive structures resembling NFTs (Supplementary Fig. 4B, Supplementary Movie 4). Similarly, we found some cells harbouring intracellular granular pSyn inclusions enveloped by CoM (Fig. 2C, D, Supplementary Movie 5). Notably, some CoM lacked AT8 tangles (Supplementary Fig. 4A) or pSyn aggregates.

We next examined the distribution of the P-Ser396 pTau (PS396), a preferred site of GSK-3 β phosphorylation activated by phospho-AKT1 (S473) [81], in combination with the S202 and S205 phosphorylated forms of tau labelled by AT8, 4G8 and Iba1 staining in IHC multiplex chromogenic experiments (*n* = 4 AD). We found relative heterogeneity of PS396 and AT8 tangles and neurites across samples, with some samples showing a distinct ratio of PS396 to AT8 loading (Fig. 2E, F). We observed single stained neurons, AT8 + or PS396 + , and double stained AT8 + PS396 + . The plaque microenvironment defined by 4G8 staining and Iba1 PaM showed a severe tau pathology, with both in AT8 and PS396 labelled swollen structures and often double-positive (Fig. 2E, F). Groups of microglia reminiscent of CoM were also found surrounding neurons with double-positive tangles (Fig. 2F). To understand the compartmentalisation of neurodegenerative processes around Aβ plaques, we used multiplex chromogenic labelling for PS396 Tau, 4G8 and glial fibrillary acidic protein (GFAP), to label PaM astrocytes that were shown to form nets [11]. We found that astrocytes frequently encapsulate PS396 + structures and Aβ plaques (Fig. 2G).

**Fig. 3:**
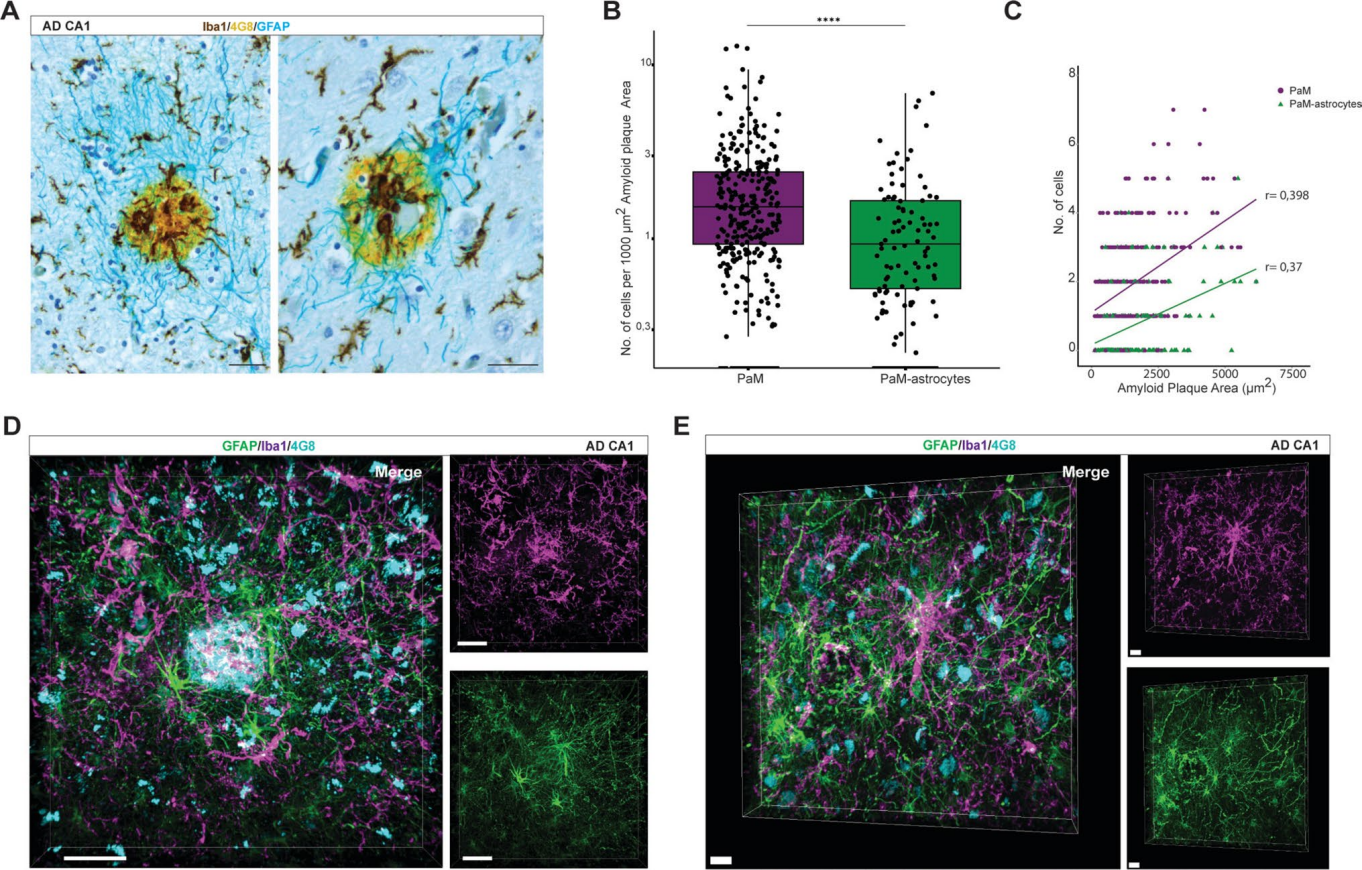
PaM and CoM show a distinct astrocytic surrounding. **A** 3-plex chromogenic IHC of Iba1 (DAB, brown), 4G8 (yellow) and GFAP (teal) showing PaM astrocytes forming polarised branches in a net-like structure (case #36). **B** Quantitative 2D analysis of PaM and PaM-astrocytes attached per Aβ plaques area. **C** The number of PaM and PaM astrocytes shows a positive correlation with the area of Aβ plaques (respectively, *r* = 0.398 and *r* = 0.37, **** *P* < 0.0001) **D** 3D confocal stack showing PaM (Iba1, magenta) surrounded by reactive astrocytes (GFAP, green) in direct proximity and forming together a RGN around Aβ plaques (4G8, cyan) (AD case #25). **E** Astrocytes (GFAP, green) do not form a polarised net around CoM (Iba1, magenta) (AD case #25). Scale bars: **A** 30µm; **D** 50µm; **E** 20µm

We then investigated in more detail the relationship of PaM and CoM with surrounding astrocytes, whose morphological changes may indicate a specific interplay with microglia. Using chromogenic multiplex IHC, for Iba1, GFAP and 4G8 (*n* = 5 CTL, 8 AD), we quantified the number of PaM and PaM-associated astrocytes (PaM astrocytes), those polarised around PaM and forming a net, associated with Aβ plaques (*n* = 391) in the CA1 and measured the corresponding plaque area (Fig. 3A–C). As expected, more PaM than PaM astrocytes were attached to Aβ plaques per area (Fig. 3B). We found a positive correlation between CA1 plaque area, the number of PaM (*r* = 0.398, ***) and the number of astrocytes forming a net (*r* = 0.37, ***) (Fig. 3C) with a similar trend as in the cortex [11]. In total, 26.1% of Aβ plaques were encapsulated by both PaM and PaM astrocytes, 22% of Aβ plaques smaller than 2500 µm^2^, 53.3% of Aβ plaques larger than 2500 µm^2^. With this approach, there was no evidence of CoM encapsulation by astrocytes. Using 3D confocal microscopy, we compared the distribution and morphology of the astrocytes surrounding PaM or CoM. Around core Aβ plaques (4G8), hippocampal PaM (Iba1) were often surrounded by an outer sphere of hypertrophic and polarised astrocytes (GFAP, PaM astrocytes), as previously observed in AD cortical brain samples (Fig. 3D), but also in APP mouse models [11]. However, we found that astrocytes surrounding CoM ( CoM astrocytes) mainly exhibited a dysmorphic phenotype, with thin and irregular branches, lacking polarisation towards the CoM and showing lower reactivity, here assessed by GFAP intensity (Fig. 3E, Supplementary Fig. 4B). Some GFAP processes were found invading CoM boundaries suggesting a possible interaction at subcellular level different from that around Aβ plaques (Supplementary Movie 4).

### Digital spatial profiling of 147 proteins highlights core differences between PaM and CoM and their microenvironment

**Fig. 4:**
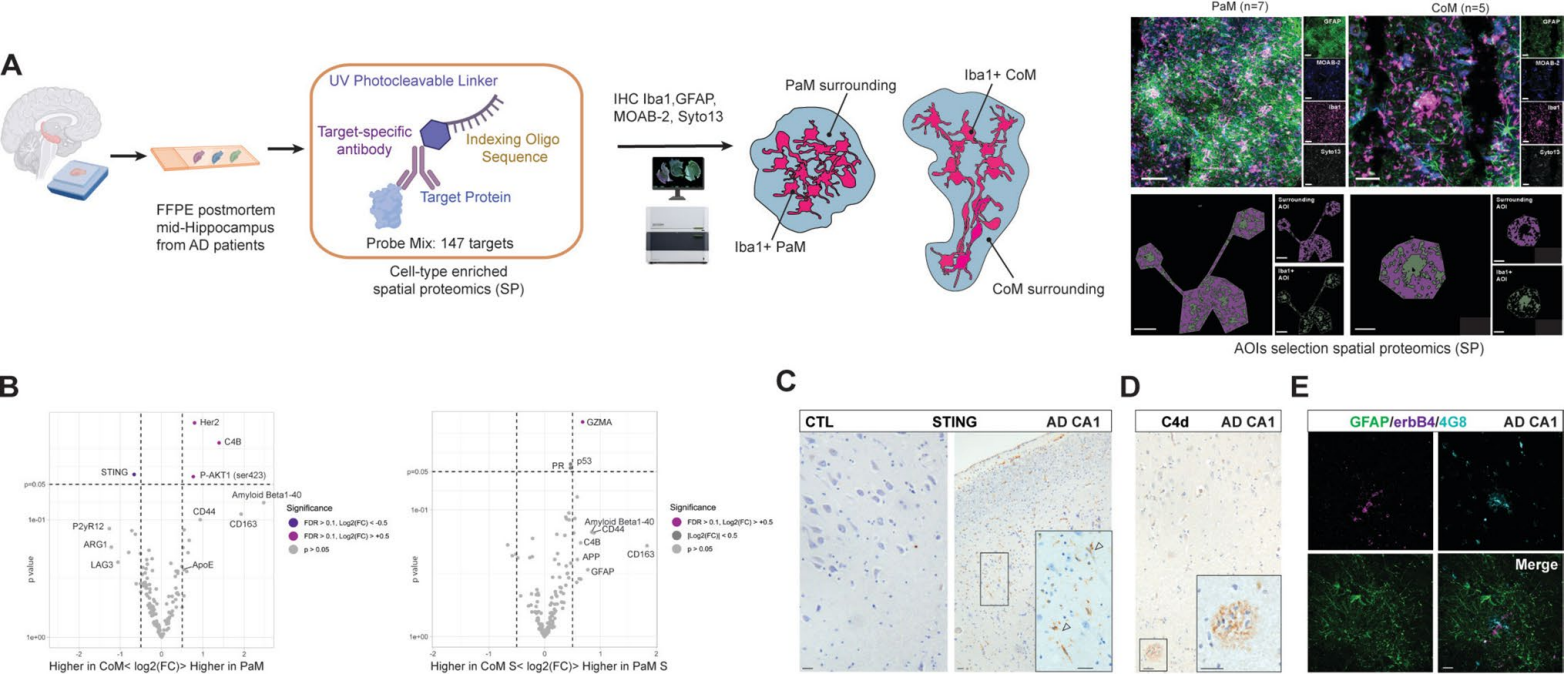
Differentially expressed proteins between PaM and CoM and their direct microenvironment. **A** Cell-type enriched spatial protein profiling workflow. One slide with 5µm-thick FFPE hippocampal sections from three AD patients (cases #49, #50 and #52) was processed for SP using Nanostring GeoMx DSP technology. SP AOIs of PaM and CoM were selected based on Iba1-segmented areas. The remaining Iba1-negative area was used to assess the surrounding of PaM and CoM. **B** Volcano plots of DEPs in microglia PaM vs. CoM (left) and between their, respectively, surrounding (right). AOIs of Thresholds of Log_2_FC = 0.5, *P* value = 0.05 and FDR = 0.1 are indicated in the graphs. **C**–**E** Validation of proteins found enriched in PaM, CoM and their surroundings. **C** STING expression (DAB, brown) in microglia is upregulated in AD vs. CTL and found in various microglia population among them, microglia in CA1 PL seemingly aggregated in a CoM-like conformation around neurons (cases #14 and #36). **D** C4d (DAB, brown) is enriched in plaques vicinity in case #36. **E** 3D confocal images show erbB4 (magenta) expression in PaM (case #45). Scale bars: **A** 100 µm; **C**–**E** 30 µm

To delve deeper into the molecular signatures of PaM and CoM in relation to their surrounding microenvironment in AD, we used the Nanostring GeoMx Digital Spatial Profiler (DSP) platform. We quantified 147 proteins in spatially defined CoM (*n* = 5) and PaM (*n* = 7) Iba1-positive areas and their close surrounding in FFPE hippocampal sections from three AD patients (cases #49, #50 and #52; Supplementary Table 1). Utilising fluorescent antibodies for microglia (Iba1), astrocytes (GFAP), Aβ-plaques (MOAB-2) and nucleic acid stain (SYTO™ 13), we accurately localised and identified PaM with astrocyte net and CoM. By manually delineating regions of interest for PaM and CoM in the hippocampal PL from the CA2 and CA1, we selected two AOIs: the Iba1-positive area and its immediate surrounding (Fig. 4A). After negative probes normalisation, only a limited number of DEPs reached significance FDR > 0.1, |Log_2_ fold change (FC)|> 0.5, *p*_adj_ < 0.05) between PaM and CoM and between their respective surroundings (Fig. 4B, Supplementary Excel 1). Her2 (receptor tyrosine-protein kinase erbB2), complement component 4B (C4B), and protein kinase B (Phospho-AKT1, S473) were enriched in PaM compared to CoM while stimulator of interferon genes (STING) exhibited higher levels in CoM. Granzyme A (GZMA), progesterone receptor (PR), and p53 were enriched in vicinity of PaM vs. CoM. Despite not reaching statistical significance, several proteins indicated potential differences in cellular signatures or microenvironments. Purinergic receptor P2RY12, Iba1, and microglia/immune cell-associated proteins such as arginase 1 (ARG1) or lymphocyte activating 3 (LAG3) were elevated in CoM compared to PaM. Amyloid peptides, amyloid precursor protein (APP), GFAP, CD163, CD44 and apolipoprotein E (ApoE) were found at higher levels in both PaM and PaM surroundings in coherence with our data and literature [61, 65]. We further validated some of these findings with immunohistochemistry (IHC), in FFPE and/or in PFA AD hippocampal sections. We confirmed the enrichment of STING in CoM, as well as C4d, a cofactor of C4B, and ErbB4, a co-receptor of ErbB2 [46] in PaM and PaM surroundings (Fig. 4C–E). Specifically, STING expression was elevated in AD hippocampal samples (8 AD, 7 CTL, FFPE), often associated with blood vessels and microglia-like cells in the white matter of the stratum oriens, CA3, and particularly the PL of CA1 (Fig. 4C). Often microglia aggregated around pyramidal neurons of the CA1 were STING immunoreactive. C4d staining was enriched around Aβ plaques in the hippocampus (CA3, CA1 and DG), subiculum, entorhinal and temporal cortex in a set of our samples (3 AD, 3 CTL, FFPE), validating previous observations [90] (Fig. 4D). Our 3D confocal analysis revealed the presence of some ErbB4-positive PaM encapsulated by GFAP-positive astrocytes in PFA-fixed hippocampal sections (Fig. 4E, Supplementary Fig. 5). These observations highlight distinct functional states and pathological microenvironments between PaM and CoM.

### Digital spatial transcriptomic profiling reveals distinct and complex molecular and metabolic states of PaM vs. CoM and surrounding astrocytes

**Fig. 5:**
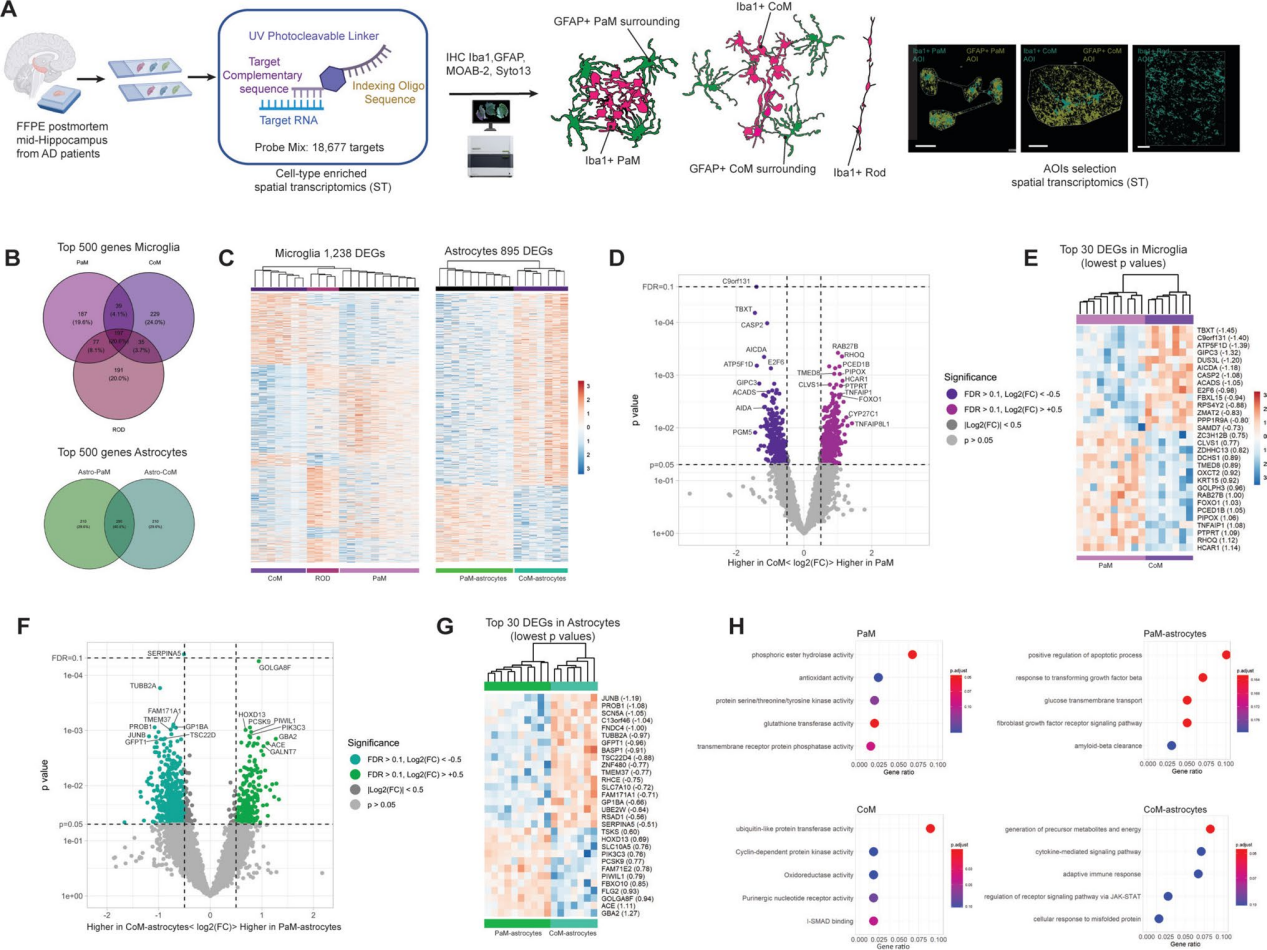
Spatial RNA profiling analysis unveil sets of differentially expressed genes between CoM and PAM and their surrounding astrocytes. **A** Cell-type enriched spatial transcriptome profiling workflow. Two slides, each with 5µm-thick FFPE hippocampal sections from three AD patients (cases #49, #50 and #52) that were cut at different depth levels of the bloc, were processed for ST using Nanostring GeoMx DSP technology. ST AOIs of PaM and CoM were selected based on Iba1-segmented areas and their surroundings based on GFAP-segmented areas. ST AOIs of Rod-shaped microglia were selected based on their bipolar morphology. **B** Venn diagrams representing overlap (total number and percentage) of top 500 genes for microglia AOIs of PaM, CoM and Rod, respectively top 500 genes for astrocyte AOIs of PaM and CoM. **C** Heatmaps of unsupervised clustering of DEGs between microglia AOIs (1,238 DEGs) and astrocyte AOIs (895 DEGs). **D** Volcano plots of DEGs in microglia AOIs of PaM vs. CoM. Thresholds of Log_2_FC = 0.5, *P* value = 0.05 and FDR = 0.1 are indicated in the graphs. **E** Heatmaps of top 30 DEGs in microglia AOIs PaM vs. CoM. Log_2_FC values are indicated in brackets for each transcript. **F** Volcano plots of DEGs in PaM vs. CoM astrocytes AOIs. **G** Heatmaps of top 30 DEGs between PaM and CoM astrocytes. **H** ORA GO of upregulated DEGs in PaM, CoM, PaM astrocytes and CoM astrocytes show distinct molecular pathways

To unravel the molecular profiles and functional states of PaM and CoM and their respective immediate surrounding cells, we used the GeoMx DSP transcriptomic platform to quantify gene expression (18,677 RNA targets) in Iba1-positive PaM and CoM, and their respective surrounding GFAP-positive astrocytes (Fig. 5A). In addition, we also profiled some Rod, defined by their elongated morphology, and sometimes clustered in CoM-Rod (Supplementary Fig. 6A). We assessed a total of 38 AOIs across two consecutive sections from three AD patient samples (cases #49, #50 and #52): 10 PaM-Iba1, 10 PaM-GFAP, 7 CoM-Iba1, 7 CoM-GFAP, and 4 Rod-Iba1 AOIs. Under UV illumination, the indexing oligonucleotide tags were collected from the probe mix and counted individually from each Iba1-positive and, their respective, GFAP-positive AOI. Briefly, all AOIs passed sequencing quality control and targets with expression in less than 5% of the segments above the LOQ were removed. The target count matrix was scaled to the geometric mean of the nuclei and normalised by the upper quartile (Q3) normalisation. A final dataset of 15,518 genes was analysed.

Comparative analysis of the 500 most highly expressed genes in each microglial and astrocyte subgroup revealed overlaps and unique gene sets. We found an overlap of 197 genes (20.6% of total genes) between all the three groups of microglia, and 290 genes (40.8%) between the two groups of astrocytes (Fig. 5B). Approximately 20% of genes were exclusive to one specific group for microglia and 30% for astrocytes. Among the highly expressed genes in all microglial subgroups were allograft inflammatory factor 1 (*AIF-1*), *CD74*, or colony-stimulating factor-1 receptor (*CSF-1R*).

In contrast, astrocytic subgroups exhibited expression of astrocyte-specific markers such as *GFAP*, aldolase C fructose-bisphosphate (*ALDOC*), and ezrin (*EZR*). We then analysed the DEGs between the microglia or astrocyte subgroups using unpaired *t* test and multiple comparison adjustment (Benjamini–Hochberg with FDR of 0.1). We identified 1,238 DEGs in our microglia dataset and 895 DEGs in our astrocyte dataset. Unsupervised clustering resulted in a clear separation of all AOIs into morphologically predefined groups for the microglia and astrocyte datasets, as shown in the heatmaps (Fig. 5C). DEGs were displayed in volcano plots for all comparisons (Fig. 5D, Supplementary Fig. 6B-C for Rod) and the top 30 DEGs identified in heatmaps (most enriched and lowest *p* value) (Fig. 5E, G, Supplementary Fig. 6D-E). When comparing PaM to CoM transcripts, we found *RAB27B*, *RHOQ*, *PCED1B*, *TMED8*, *HCAR1*, *CLVS1*, *GOLPH3*, *PTPRT*, *DCHS1*, *TNFAIP1*, *FOXO1*, *OXCT2*, and *HMCES* among the most enriched and statistically significant genes for PaM; while in CoM they were *C9orf131*, *TBXT*, *CASP2*, *AICDA*, *E2F6*, *ATP5F1D, MAP2K5*, *GIPC3*, *PPP1R9A*, *FBXL15*, *ACADS, ZMAT2*, *GSDME DUS3L*, *GDF1*, and *BLOC1S3*. Rod-shaped microglia also showed numerous DEGs compared to both CoM and PAM. *TAAR1*, *BCKDHA*, *CTNNAL1*, *EDIL3*, *RYR1*, *CALML4*, *RAX2*, *SLC20A2* and *SERPINB6* were among the top 20 most DEGs in both comparisons (Supplementary Fig. 6B-C, Supplementary Excel 1).

We then performed an over-representation analysis (ORA) for PaM and CoM DEGs. The Gene Ontology (GO) pathways over-represented in PaM revealed phosphorylation and lipid degradation activities, antioxidant and glutathione transferase and tyrosine kinase activities. Consistent with this, the ORA based on the Kyoto Encyclopedia of Genes and Genomes (KEGG) showed associations with sphingolipid signalling pathways, insulin resistance, ErbB and Wnt signalling pathways or necroptosis (Supplementary Excel 2) (Fig. 5H). The top five GO pathways for CoM were associated with ubiquitin-like protein transferase, purinergic nucleotide receptor, oxidoreductase cyclin-dependent kinase activities (Fig. 5H). ORA KEGG and Wikipathways (WP) suggested associations with transforming growth factor (TGF)-β and NF-κB signalling, among others (Supplementary Excel 2).

Next, we analysed DEGs in PaM astrocytes vs. CoM astrocytes. PaM astrocytes were associated with distinct signatures, and we found *GOLGA8F*, *PCSK9*, *HOXD13*, *PIWIL1*, *PIK3C3*, *FAM71E2*, *FLG2*, *GBA2*, *SLC10A5*, *ACE*, *FBXO10*, *ARHGAP29*, *CES1*, *TMEM273*, and *GALNT7* among the most enriched genes (Fig. 5F, G, Supplementary Excel 1). We found *SERPINA5*, *TUBB2A*, *FAM171A1*, *PROB1*, *GP1BA*, *TMEM37*, *FNDC4*, *JUNB*, *SCN5A*, *TSC22D4*, *GFPT1*, *BASP1*, and *UBE2W* among the most enriched and statistically significant genes for CoM astrocytes. We performed ORA on PaM astrocytes and CoM astrocytes DEGs. ORA GO of DEGs PaM astrocytes showed enrichment for fibroblast growth factor receptor signalling, response to TGF-β, glucose transmembrane transport, positive regulation of apoptotic process and amyloid beta clearance (Supplementary Excel 2). ORA KEGG and WP indicated activities associated with PI3K-Akt signalling pathway, cholesterol metabolism or cell adhesion molecules, fatty acid and lipoprotein transport or complement system. ORA GO highlighted the association of DEGs CoM astrocytes with metabolite and energy generation (OXPHOS), cytokine mediated pathways but also with adaptive immune response, JAK-STAT signalling and cellular response to misfolded proteins (Fig. 5H, Supplementary Excel 2). Our DSP analysis revealed specific cellular and molecular identities of PaM and CoM, and of their respective surrounding astrocytes.

### Markers of the molecular signatures of PaM, CoM and surrounding astrocytes in the hippocampus of AD patients

**Fig. 6:**
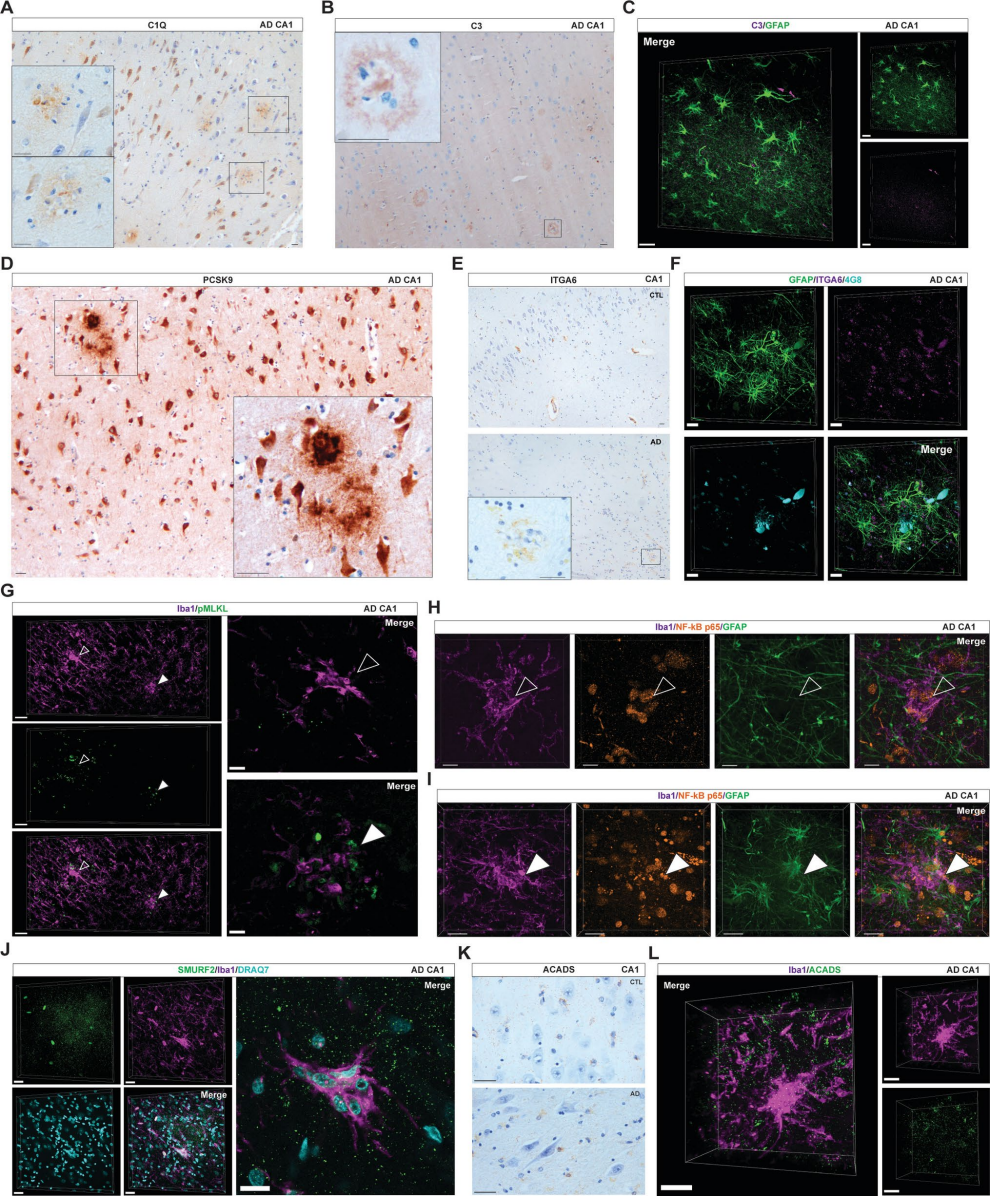
Validation of functional markers for PaM, PaM astrocytes and CoM by IHC on human post-mortem hippocampal samples. **A** C1q (DAB, brown) is expressed in the vicinity of plaques, in cells resembling microglia in CA1 AD (case #30). Some pyramidal neurons were also stained by C1q in AD. **B**, **C** C3 (DAB, brown) is also expressed close to plaques (cases #38 and #40). **C** In confocal microscopy, C3 (magenta) punctuated distribution is found within PaM astrocyte territories (GFAP, green). **D** PCSK9 (DAB, brown) which is mainly expressed by neurons is enriched in the plaque vicinity in AD (case #44). **E**, **F** ITGA6 is associated with blood vessels and plaque microenvironments (cases #14, #33 and #35). **G** IHC fluorescent high-resolution confocal 3D acquisitions of a post-mortem AD hippocampus showed an enrichment of the necroptosis marker pMLKL (green) in the vicinity of plaques (full arrows), while CoM (empty arrows) were not engulfing pMLKL + structures (left panel 3D, right panel z focal plan) (case #29). **H**, **I** NF-κB p65 (orange) nuclear translocation was found at high level in CoM (empty arrows) (**H**), in PaM (full arrows), and PaM astrocytes (**I**) in AD samples (case #25). **J** Confocal 3D images show that CoM (Iba1, magenta) express some SMURF2 (green) proteins (cases #52, # 35). **K** Chromogenic staining show the distribution of ACADS (DAB, brown) in the CTL and AD hippocampi (cases #15 and #37). **L** Confocal 3D images show that ACADS (green) expression is found in CoM (Iba1, magenta) (cases # 35). Scale bars **A**–**F**, **H**, **I**, **K**, **L** 30 µm; **G** 40 µm (low mag), 10 µm (high mag); **J** 30 µm (low mag), 15 µm (high mag)

To validate the molecular signatures identified in PaM and PaM astrocytes by our DSP analysis, we performed IHC on selected proteins that embodied an enriched pathway in FFPE or PFA samples. First, we examined the complement component 1q (C1q) (*n* = 5 AD, 5 CTL) and complement component 3 (C3) (*n* = 3 AD, 2 CTL) association with PaM and the PaM environment or PaM astrocytes. In AD, C1q was strongly distributed around plaques, and on some hippocampal neuronal cell bodies and neurites in the CA3-CA1 subfields, but also in the DG (Fig. 6A) and entorhinal cortex (Supplementary Fig. 6A), and occasionally diffuse in areas reminiscent of astrocyte domains. C1q-positive hippocampal neurons were also found in CTL (Supplementary Fig. 7A). Similarly, C3 staining exhibited lower overall expression, primarily labelling the core and corona of Aβ plaques in AD (Fig. 6B, Supplementary Fig. 7B). Using high-resolution 3D confocal imaging, we observed C3 expressed within GFAP-positive PaM astrocytes territories, in cell bodies but also along processes (Fig. 6C). We then analysed the distribution of Proprotein convertase subtilisin/kexin type 9 (PCSK9) (*n* = 3 AD, 2 CTL) and Integrin subunit alpha 6 (ITGA6, also known as CD49f [6]) (*n* = 5 AD, 5 CTL), associated with the PaM astrocytes and PI3K-Akt pathways in our previous analysis. In FFPE sections, we observed a PCSK9 expression in neurons both in CTL and AD (Supplementary Fig. 7); however, in AD samples, PCSK9 staining was also associated with plaque microenvironments and with cells resembling PaM astrocytes in hippocampus and entorhinal cortex (Fig. 6D, E, Supplementary Fig. 7). ITGA6 was predominantly distributed along blood vessels (BV) in CTL, while in AD, expression around BV was weaker and occasionally observed near plaques, apparently associated with PaM astrocytes. We confirmed a low expression of ITGA6 in the cell bodies and processes of PaM astrocytes with confocal microscopy (Fig. 6F). We then investigated the association of the PaM microenvironment with necroptosis identified through pathway analysis. We found, in bright-field and 3D confocal images, a clear enrichment of phosphorylated mixed lineage kinase domain-like protein (pMLKL), part of the necrosome complex, in PaM vicinity but no staining in CoM (Fig. 6G). PMLKL was frequently concentrated in vacuole-like accumulations within the cytoplasm of numerous pyramidal neurons in AD samples and, to a lesser extent, in CTL as previously described [42].

DSP analysis suggested that CoM was involved in different biological processes than PaM. To further elucidate these processes, we performed co-immunostaining against Iba1, GFAP and NF-κB -p65 in PFA sections revealing strong expression and translocation of NF-κB -p65 into CoM nuclei by confocal microscopy (Fig. 6H). The staining was not exclusive to CoM, but also detected in the surrounding microenvironment, without being predominantly expressed in astrocytes. In contrast, PaM and PaM astrocytes displayed heterogeneous NF-κB -p65 staining patterns (Fig. 6I). ORA analysis revealed strong ubiquitin ligase activity in CoM. Confocal microscopy analysis of E3 ubiquitin-protein ligase SMURF2 distribution in AD PFA-fixed samples revealed expression in CoM, although not significantly enriched compared to the surrounding microenvironment (Fig. 6J). Acyl-CoA dehydrogenase (ACADS) is associated with fatty acid oxidation and high metabolic activity. In CTL samples, ACADS was expressed in astrocytic cells in the temporal cortex, CA4 hippocampus and CA3-CA1 PL. In AD, astrocytes retained ACADS expression, but so did some microglia surrounding pyramidal neurons. 3D confocal analysis confirmed the co-distribution of ACADS in the CoM (Fig. 6K, L).

### Macrophage and T cell association with PaM and CoM

**Fig. 7:**
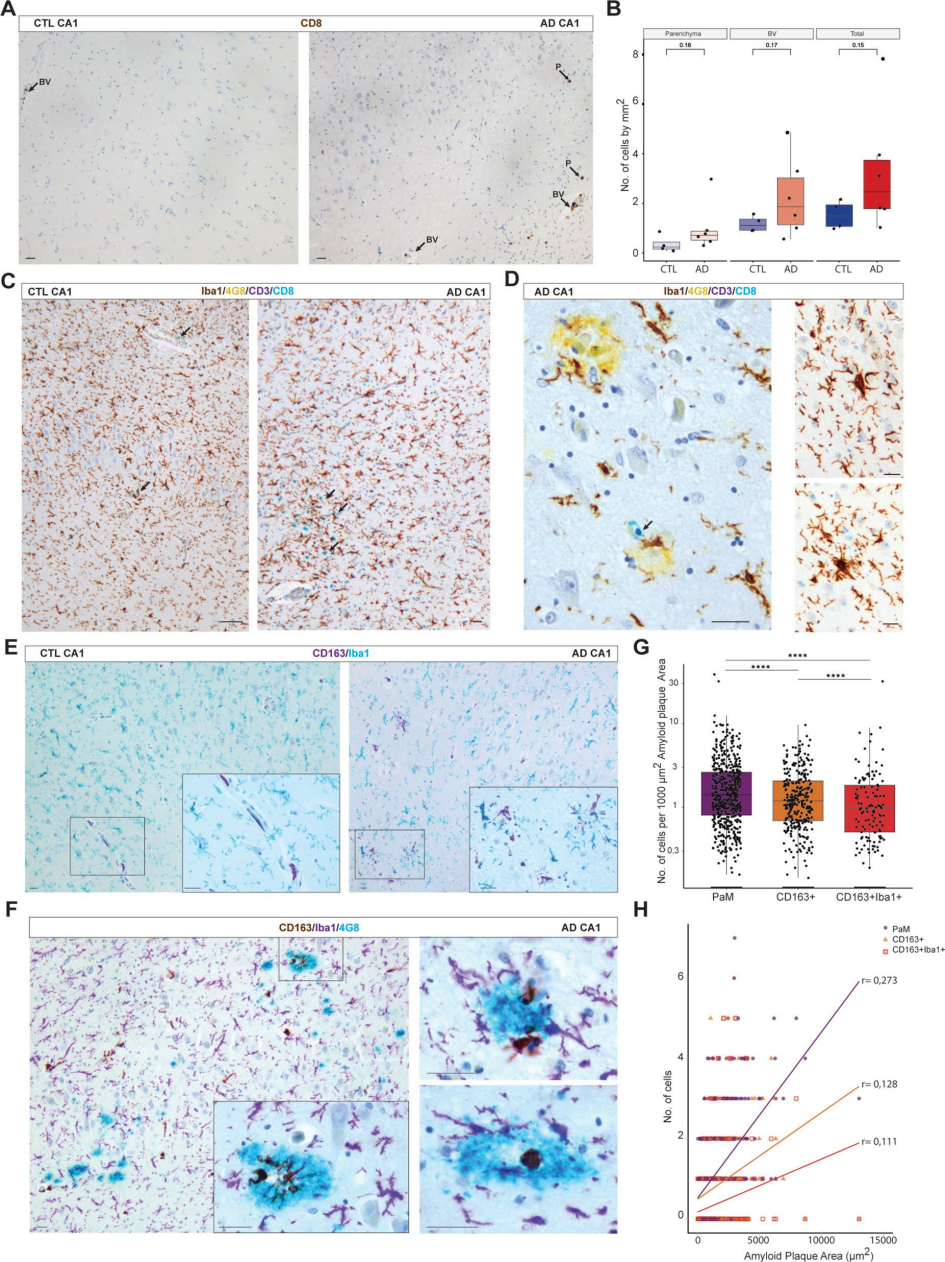
Multiplex chromogenic IHC reveals the involvement of infiltrating immune cells in PaM and CoM. **A** CD8 + (DAB, brown) are often found in BV and, however, very infrequently in the brain parenchyma of CTL (case #7) and AD hippocampi (case #49). **B** Quantification of CD8 + cells in CTL and AD hippocampal samples shows a slight increase but no statistical differences between CTL and AD samples (CTL *n* = 4, AD *n* = 6). **C**, **D** 4-plex chromogenic IHC for Iba1 (DAB, brown), CD3 (purple), 4G8 (yellow) and CD8 (teal) showed that most T cells found in our samples were CD8 + (black arrows), located within BV in age-matched CTLs hippocampi CA1 (**C**, left panel) or sometimes infiltrated into the parenchyma (**C**, right panel). **D** A minority of CD8 + (teal) were found near plaques but were not mixed with PaM or were never associated with CoM (**C**, **D**; cases #15, #40, #30 and #44). **E** Double staining for CD163 and Iba1 showed CD163 + cells (purple) covering BV and Iba1 + ramified cells (teal) regularly distributed in the parenchyma in age-matched CTL hippocampi (left panel, CA1). In AD cases, Cd163 + cells were often found accumulating with PaM Iba1 + (right panel, CA1). Cd163 + Iba1 + (dark blue) cells were frequently found near PaM and blood vessels in AD (cases #8 and #30). **F** 3-plex chromogenic IHC of CD163 (DAB, brown), Iba1 (purple) and 4G8 (teal) confirmed the infiltration of CD163 + cells within the PaM in AD hippocampus (case #37). **G** Quantitative 2D analysis of PaM, CD163 + and CD163 + Iba1 + attached per Aβ plaques area. **H** The number of PaM, CD163 + and CD163 + Iba1 + show a positive correlation with the area of Aβ plaques (respectively, *r* = 0.273, **** *P* < 0.0001; *r* = 0.128, ** *P* < 0.01 and *r* = 0.111, * *P* < 0.05). Scale bars: **A**–**F** 30 µm

To determine whether infiltrating immune cells contribute to the PaM or CoM molecular markers and composition, we next examined the distribution of T cells and CNS-associated macrophages in CTL and AD hippocampal samples. Indeed CoM exhibited a cellular organisation reminiscent of nodules observed in various brain infections, often containing T cells [80, 95]. First, we quantified the number of CD8 + T cells, which have previously been detected near Aβ plaques in AD brain samples, in CTL and AD hippocampi (4CTL, 6 AD). CD8 + T cells were mainly found in BV and less frequently in hippocampal parenchyma (Fig. 7A). CD8 + T cells were slightly increased in AD without reaching significance (Fig. 7B). To further characterise the properties and distribution of CD8 + T cells, we used multiplex chromogenic IHC to stain CD3 in combination with CD8, together with 4G8 for Aβ plaques and Iba1 for microglia in some CTL and AD hippocampal samples (CTL *n* = 3; AD *n* = 6). With this approach, we detected only single positive cells, mainly CD8 + and sometimes CD3 + (Fig. 7C, D). In CTL hippocampus, CD8 + cells were strongly localised in the BV, with occasional presence in the parenchyma, close to microglia. In AD, we again observed a slight increase in parenchymal CD8 + cells, sometimes infiltrating in hotspots, but with very little to no association with PaM, Aβ plaques or CoM (Fig. 7D). Similar observations were made in the entorhinal and temporal cortices of AD samples (Supplementary Fig. 8A).

We next examined the presence of macrophages as DSP revealed shared transcripts between PaM and peripheral immune cells. As CD163 was one of the proteins enriched in PaM and the PaM surroundings, we investigated its distribution using two multiplex chromogenic IHC strategies. When we coupled Iba1 staining with CD163, we observed, in CTL samples (*n* = 3), that CD163 + perivascular cells were regularly distributed along the BV, while Iba1 + cells populated the parenchyma. Few Iba1 + CD163 + cells were detected lining the capillaries (Fig. 7E). In contrast, AD samples (*n* = 4) exhibited a notable increase in CD163 + cells within the parenchyma, often regrouped among PaM-like accumulations with several CD163 + cells being double positive for Iba1(Fig. 7E). CD163 + Iba1 + were abundant in the white matter of the stratum oriens. To further understand their relationship with Aβ plaques, we combined CD163, Iba1 and 4G8 staining on CTL and AD samples (5 CTL, 8 AD). We found a recurrent co-distribution of CD163 + , Iba1 + and Iba1 + CD163 + around Aβ plaques in the hippocampus (Fig. 7F), entorhinal and temporal cortices (Supplementary Fig. 8B). Using digital pathology, we have quantified the association of Iba1 + , CD163 + , and CD163 + Iba1 + with Aβ plaques and their respective areas in the CA1 of AD cases. The number of Iba1 + PaM associated with plaque area was significantly higher than CD163 + and CD163 + Iba1 + which were the less frequent. We found a positive correlation between CA1 plaque size and the number of PaM (*r* = 0.273, ****) but low positive correlation for CD163 + (*r* = 0.128, **) and CD163 + Iba1 + (r = 0.111, *).

## Discussion

Our investigation provides a comprehensive dissection of the molecular identities underlying microglia-encapsulated neurodegenerative microenvironments, particularly evident in the aggregation patterns observed in the hippocampi of AD patients**.** Using a combination of spatial protein and transcriptomic profiling, brightfield chromogenic multiplex IHC and 3D confocal and STED microscopy, our findings highlight the multiple identities of microglia and astrocytes in AD and their potential impact on the deterioration of the hippocampus, and the brain.

**Fig. 8:**
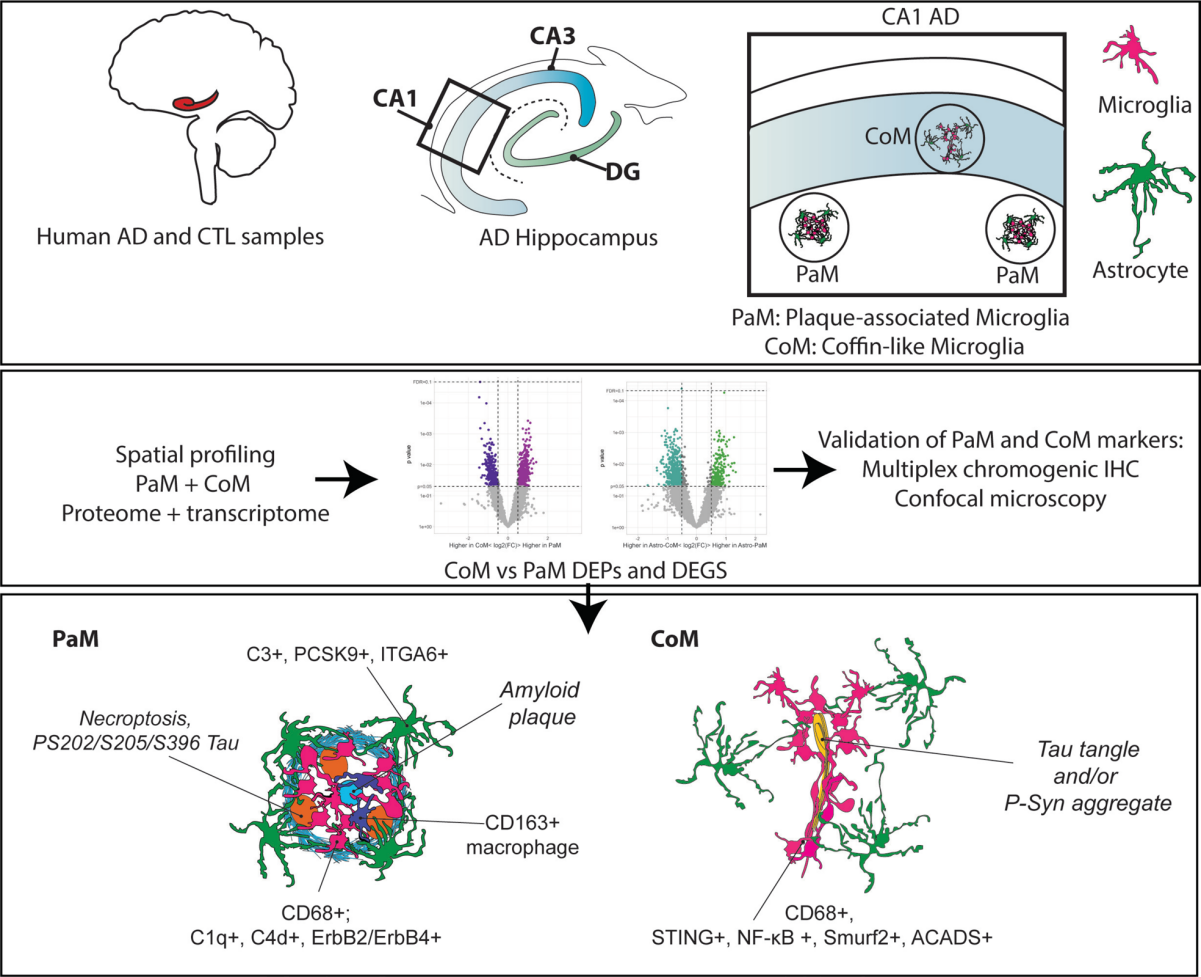
Graphical summary showing the differential signatures of PaM and CoM in the human AD hippocampus

We describe the distinct structural and functional attributes of PaM and their surrounding cells, the astrocytes, as well as a newly identified type of microglia accumulation termed CoM, enriched in AD CA1. While CoM resemble microglial nodules found in multiple sclerosis or brain infections [13, 21, 76, 80, 94, 95], their identity appeared to differ in many ways (Fig. 8). CoM exhibit a unique pyramidal-like structure composed of densely packed Iba1-positive cells encircling diseased or dying cells and independent of Aβ plaques. CoM were also occasionally found in DLB CA1 hippocampi but were absent in other brains regions of AD and DLB patients investigated in this study including prefrontal, temporal and entorhinal cortices. The selective enrichment of CoM in CA1 suggests localised vulnerability and potential disruption to hippocampal integrity and circuitry. This finding highlights CoM as a potential contributor to the progression of neurodegeneration in the hippocampus. Our data also suggest a strong, albeit non-systematic, association of CoM with tau and synuclein pathology. These findings align with our previous analyses of individual microglial morphological changes, which demonstrated a robust relationship between ‘disease morphology clusters’ and tau pathology in the AD hippocampus [22]. Notably, tau pathology was more severe in CA1 compared to other hippocampal subfields, suggesting an increased sensitivity or predisposition of CA1 microglial populations to pathological stimuli, potentially triggering the formation of CoM. To our knowledge, CoM have not been described in any mouse models of AD, amyloid or tau, but a study by Beckman and colleagues showed a clear association between engulfing microglia and tangle-positive neurons specifically in the CA1 subfield in macaques injected with a dual tau mutation (P301L/S320F) arguing for a specific vulnerability of this neuronal population, at least in primates [7]. Although sharing some similarities with PaM, such as the expression of CD68, CoM differ in their molecular machinery, particularly illustrated by their association with intraneuronal tau tangles or pSyn aggregates. While CoM express a distinct repertoire of markers associated with protein degradation, STING, TGF-β, NF-κB signalling, p53, and the SMAD ubiquitin–proteasome system (UPS), they notably lack markers indicative of T cell infiltrations. The CoM signature appears to be distinct from AD-associated microglial signatures identified in amyloid mouse models, such as disease-associated microglia (DAM) [40], microglial neurodegenerative phenotype (MGnD) [43], activated response microglia (ARM) [25], interferon response microglia (IRM) [25], or WAM [76]. In our study, we did not find strong evidence for the involvement of CD8 + T cells, which were not significantly increased in AD compared to CTL and were not found near CoM or PaM. Furthermore, astrocytes surrounding CoM appeared dysmorphic without showing a specific disease-signature. The presence of CoM in the CA1 region in DLB cases, suggests a shared pathogenic mechanism underlying hippocampal neurodegeneration across diseases [105]. Our results hint about a relationship between the disease state of pyramidal neurons, maturation of tau tangles and CoM [59]. However, elucidating the precise signalling cues driving CoM formation and their impact on disease severity requires further investigations.

Our study reveals complex interactions between PaM, PaM astrocytes, and CNS-associated macrophages in shaping the acute neurodegenerative microenvironment surrounding Aβ plaques in the hippocampi of AD patients. Notably, our findings are not restricted to the hippocampus, as we observed similar PaM signatures in other brain regions such as the entorhinal and temporal cortices. Our data confirmed the organisation of PaM and PaM astrocytes in RGN [11] in the hippocampus, with astrocytes enclosing PaM, themselves accumulating towards the core of the plaque. Our data show a positive correlation between both the number of PaM or PaM astrocytes with the size of the Aβ plaques, with a higher positive value for PaM. This suggests a sequential formation of the RGN with an early accumulation of PaM prior to the polarisation of surrounding astrocytes into a net. Furthermore, our investigation highlights the heterogeneity within the PaM population. Specifically, PaM (Iba1 + only) coexist with infiltrating CD163 + cells and double-positive for Iba1 and CD163 cells. CD163 + cells appear to be perivascular macrophages, whereas Iba1 + CD163 + cells may represent differentiated macrophages or microglia. Our findings are consistent with other recent studies showing higher CD163 expression in AD brain parenchyma [61, 65]. Our quantitative data suggest that the association of CD163 + and Iba1 + CD163 + with PaM and plaques is less frequent than the ones of PaM and PaM astrocytes and may be mediated by specific triggers or pathways. It should be noted that the Iba1 + PaM signature found in our SP analysis may partially overlap with that of the Iba1 + CD163 + cells attached to plaques, which also illustrates a certain heterogeneity between individual PaM but also CoM to be considered. Nevertheless, the definitive role of CD163 + macrophages at the plaque level is not defined by our study, but they may be prominent in orchestrating C1q production by PaM. De Schepper et al. have recently shown that secreted phosphoprotein 1 (SPP1/osteopontin) is mainly produced by perivascular macrophages and, when secreted, triggers C1q production by microglia and their phagocytic activity in an AD mouse model [20]. In the study, this mechanism was mainly described as a driver of abnormal synaptic pruning in AD. A similar increase in phagocytic activity may be relevant to Aβ clearance at the plaque level. Furthermore, our study uncovers the concomitant enrichment of C1q, C3, C4b and d, confirming what others have previously observed in the AD human brain [5, 24, 70, 74, 90]. Here, DSP and IHC image analysis, helped to associate C1q and C4 production with microglia [23] and C3 with astrocytes [48]. The C3 produced by astrocytes may play a dual role in limiting the spread of amyloid or in neurodegeneration. Indeed, Wyss-Coray et al. have shown that expression of soluble complement receptor-related protein y (sCrry), an inhibitor of C3 activation, in the APP/PS1 mouse model leads to a decrease in PaM activation, a 2–threefold increase in amyloid load and a higher accumulation of degenerating neurons [106]. However, C3 is also associated with a neurotoxic signature of astrocytes [10, 48]. C1q, C3 and C4 are also associated with Wnt signalling [62], metabolic disorders, and insulin resistance pathways found in our ORA of PaM DEGs, or cholesterol metabolism found in our ORA of PaM astrocytes DEGs [82]. The classical complement cascade has been highlighted as part of the Aβ plaque-induced genes (PIGs) by a study using a different spatial profiling approach in the APP NL-G-F mouse model, showing some overlap between human AD cellular responses and mouse models [17]. Moreover, PaM astrocytes express both PCSK9 and ITGA6, which, along C3, may influence microglial functions. PCSK9, known to regulate low-density lipoprotein (LDL) cholesterol levels, is expressed by hippocampal neurons in control and AD conditions, but also by PaM astrocytes in AD samples. Elevated PCSK9 RNA and protein levels have been reported in cortical tissue of AD patients compared to CTL [66], and its increase in cerebrospinal fluid (CSF) of AD patients correlates with ApoE, Aβ42 and pTau. PCSK9 may have multiple local effects on amyloid clearance, lipid metabolism and neuroinflammation. Briefly, PCSK9 influences beta-site APP-cleaving enzyme 1 (BACE1) levels [37]. It has a direct effect on astrocyte cholesterol metabolism, increasing its synthesis and decreasing ApoE-HDL (high-density lipoprotein) derived cholesterol uptake in vitro [64]. In the same study, authors found that it promotes an Aβ neurotoxic effect on neurons. PCSK9 may also play a role in the pro-inflammatory response of microglia, as shown in vitro [102]. Genetic deletion of PCSK9 in the 5xFAD mouse model attenuates Aβ burden, microglial activation, astrocytosis, and cognitive deficits, highlighting its potential as a therapeutic target in AD [100]. PCSK9 is also involved in modulating the pro-inflammatory state of macrophages and the inflammasome response [38, 72, 103]. The role of the astrocyte-secreted PCSK9 at the level of Aβ plaques may then be critical in altering local cholesterol metabolism, glial and immune cell states. In addition to PCSK9, our study identifies ITGA6, a cell surface receptor implicated in blood–brain barrier maintenance and inflammation [16], as being expressed by PaM astrocytes. ITGA6, also called CD49f, is expressed by a subpopulation of human iPSC-derived astrocytes in vitro [6, 99] and is shown here in RGN astrocytes, possibly related to their reactive profile. We also report ErbB2-ErbB4-positive PaM and observed erbB4-positive astrocytes in AD hippocampus. ErbB signalling is involved in astrocyte reactivity [15] and erbB4 has been found to be expressed in the vicinity of plaques in association with neuronal NRG1 [14]. ErbB signalling may also play a role in the activation state of both microglia and macrophages [49, 79].

The encapsulation of plaques by microglia, perivascular macrophages, and surrounding polarised astrocytes orchestrates a neurodegenerative micro-environment involved in disease progression [11]. We postulate that prolonged exposure of neurons trapped in RGN to molecules secreted by PaM and PaM astrocytes is likely to accelerate their degeneration. As evidenced by the high levels of P-Akt1 (S423) [18], the presence of swollen neurites and severe tau hyperphosphorylation observed in the hippocampal and cortical RGN [96]. Additionally, the detection of markers indicative of necroptosis further underscores the detrimental impact of this neurodegenerative niche. In this study, however, we do not observe any association between T cell infiltration and RGN or CoM [58]. The distinct molecular signature of rod-shaped microglia, which can also regroup in a CoM-like structure, confirms a heterogeneity of states in AD and emphasises the diversity of microglial responses.

## Conclusions

Our study identifies distinct microglial aggregates, PaM and CoM, in the AD hippocampus, each with unique pathological and molecular signatures. Using deep spatial profiling and advanced microscopy, we reveal that CoM are associated with tau and α-synuclein pathology and protein degradation, while PaM are associated with amyloid deposition, complement pathways, and immune cell infiltration. We also identify new astrocyte signatures that shape the peri-plaque microenvironment. These findings elucidate complex glial-immune interactions driving neuroinflammation and hippocampal degeneration, providing a foundation for novel therapeutic strategies targeting glial and immune cells to mitigate AD progression.

Our DSP analysis was conducted on a limited number of samples and brain regions obtained from fixed post-mortem human brains. This limited sampling may not fully capture the heterogeneity of AD pathology, PaM and CoM, across different disease stages and brain regions. Moreover, the DSP approach, while powerful, is not without its limitations. It may not fully capture the specific molecular signatures of individual cell types. To partially address this issue, we performed a general validation of identified markers using IHC on a larger set of samples using different methodological approaches.

## Supplementary Information

Below is the link to the electronic supplementary material.

Supplementary file1 (PDF 1488 KB)

Supplementary file2 (MOV 9451 KB)

Supplementary file3 (MOV 30461 KB)

Supplementary file4 (MOV 20611 KB)

Supplementary file5 (MP4 1396 KB)

Supplementary file6 (MOV 5434 KB)

Supplementary file7 (XLSX 201 KB)

Supplementary file8 (XLSX 112 KB)

## Data Availability

Data are provided within the manuscript or supplementary information files. Raw DSP data are available on request.

## Abbreviations

AA: Amyloid angiopathy
ACADS: Acyl-CoA dehydrogenase
AD: Alzheimer’s disease
ADRD: Alzheimer’s disease-related dementias
AIF1: Allograft inflammatory factor 1
ALS: Amyotrophic lateral sclerosis
ALDOC: Aldolase C fructose-bisphosphate
AOI: Areas of illumination
AP: Alkaline Phosphatase
ApoE: Apolipoprotein E
APP: Amyloid precursor protein
ARG1: Arginase 1
ARM: Activated response microglia
Aβ: Amyloid-β
BACE-1: Beta-site APP-cleaving enzyme 1
CA: Cornu ammonis
CC1: Cell conditioning 1
CC2: Cell conditioning 2
CD68: Cluster of differentiation 68
CM: ChromoMap
CNS: Central nervous system
CoM: Coffin-like microglia
CoM-Rod: CoM formed predominantly by rod-shaped microglia
CSF: Cerebrospinal fluid
CSF-1R: Colony-stimulating factor-1 receptor
CTL: Control
CTX: Cortex
C4B: Complement component 4B
C1q: Complement component 1q
C3: Complement component 3
DAB: 3, 3′-Diaminobenzidine
DAM: Disease-associated microglia
DEGs: Differentially expressed genes
DEPs: Differentially expressed proteins
DG: Dentate gyrus
DLB: Dementia with Lewy bodies
DSP: Deep spatial profiling
ENT: Entorhinal
EZR: Ezrin
FC: Fold change
FDR: False discovery rate
FFPE: Formalin fixed paraffin embedded
GFAP: Glial fibrillary acidic protein
GWAS: Genome-wise association studies
GO: Gene ontology
GZMA: Granzyme A
HDL: High-density lipoprotein
HIER: Heat induced epitope retrieval
HRP: Horseradish peroxidase
Iba1: Ionized calcium-binding adaptor molecule 1
IHC: Immunohistochemistry
IRM: Interferon response microglia
ISH: In situ hybridization
ITGA6: Integrin subunit alpha 6
KEGG: Kyoto Encyclopedia of Genes and Genomes
LAG3: Lymphocyte activating 3
LDL: Low-density lipoprotein
LOAD: Late-onset AD
LOQ: Limit of quantitation
MGnD: Microglial neurodegenerative phenotype
MS: Multiple sclerosis
NDD: Neurodegenerative disease
NFT: Neurofibrillary tangles
NGS: Next Generation Sequencing
ORA: Over-representation analysis
PaM: Plaque-associated microglia
PBS: Phosphate-buffered saline
PCSK9: Proprotein convertase subtilisin/kexin type 9
PD: Parkinson's disease
PFA: Paraformaldehyde
PIG: Amyloid-β plaque-induced genes
PL: Pyramidal layer
PMD: Post-mortem delay
pMLKL: Phosphorylated mixed lineage kinase domain-like protein
PR: Progesterone receptor
pSyn: Phosphorylated α-synuclein
pTau: Hyperphosphorylated tau
Q3: Upper quartile
RGN: Reactive Glia Net
RT: Room temperature
sCrry: Soluble complement receptor-related protein y
SP: Spatial proteomics
SPP1: Secreted phosphoprotein 1
ST: Spatial Transcriptomics
STED: Super resolution stimulated emission depletion microscopy
STING: Stimulator of interferon genes
TDP-43: TAR DNA-binding protein
TGF: Transforming growth factor
TR: Thiazine red
TREM2: Triggering receptor expressed on myeloid cells 2
UMI: Unique Molecular Identifier
UPS: Ubiquitin–proteasome system
UV: Ultraviolet
WAM: White matter-associated microglia
WP: Wikipathways
3D: Three dimension
