# Supplementary material for: Microglia aggregates define distinct immune and neurodegenerative niches in Alzheimer's disease hippocampus
Source: Acta Neuropathol. 2025 Feb 15;149(1):19. doi: 10.1007/s00401-025-02857-8 (PMC11829914; doi:10.1007/s00401-025-02857-8)
Supplement: Supplementary file 1 — Supplementary file1 (PDF 1488 KB) [file 401_2025_2857_MOESM1_ESM.pdf]

Microglia aggregates define distinct immune and neurodegenerative niches in Alzheimer's disease hippocampus.

Sonja Fixemer[1,2]*, Mónica Miranda de la Maza[1,2,3,4]*, Gaël Paul Hammer[2,3], Félicia Jeannelle[2,3], Sophie Schreiner[2,3], Jean-Jacques Gérardy[2,3], Susana Boluda[5,6], Dominique Mirault[7], Naguib Mechawar[7], Michel Mittelbronn[1,2,3,4,8,9], David S. Bouvier[1,2,3] .

Author affiliations:

[1] Luxembourg Centre for Systems Biomedicine (LCSB), University of Luxembourg, Belval, Luxembourg.

[2] Luxembourg Centre of Neuropathology (LCNP), Dudelange, Luxembourg.

[3] Laboratoire national de santé (LNS), National Center of Pathology (NCP), Dudelange, Luxembourg.

[4] Department of Cancer Research (DOCR), Luxembourg Institute of Health (LIH), Luxembourg.

[5] Department of Neuropathology, Pitié-Salpêtrière Hospital, AP-HP Sorbonne University, Paris, France

[6] Institut du Cerveau - Paris Brain Institute - ICM, Inserm U1127, CNRS UMR7225, APHP, Sorbonne University, Pitié-Salpêtrière Hospital, Paris, France.

[7] Douglas Mental Health University Institute, Department of Psychiatry, McGill University, Montreal, Quebec, Canada.

[8] Department of Life Sciences and Medicine (DLSM), University of Luxembourg, Esch-sur-Alzette, Luxembourg.

[9] Faculty of Science, Technology and Medicine (FSTM), University of Luxembourg, Esch-sur-Alzette, Luxembourg.

* : equally contributing authors

Correspondence to: Dr. David S. Bouvier

Laboratoire national de santé (LNS)

1, Rue Louis Rech

L-3555 Dudelange

Email: david.bouvier@lns.etat.lu ;david.bouvier@ext.uni.lu

Supplementary Fig.1 Super resolution of PaM and CoM present in AD and DLB hippocampi. (A, B) STED 3D acquisitions of PFA-fixed AD hippocampal sections immunostained against Iba1 (magenta) and Aβ plaques (4G8, green) reveal the morphology of (A) PaM and (B) CoM. PaM and CoM are depicted in 3D (upper panels) and 2D images throughout the z axis (lower panels) (case #52)). (C-D) High-resolution confocal acquisitions of *post-mortem* hippocampal tissue from DLB patients immunostained against microglia (Iba1, magenta) and nuclei (DRAQ7[TM], white). (C) PaM (case #20) and (D) CoM (case #21) are often present in DLB hippocampi. Scale bars: (A, C) 20µm; (B) 10µm; (D) 30µm.

Supplementary Fig.2 Nuclei are often encapsulated in CoM. The 3D stack allows to follow through focal plan (each 1µm) the complete encapsulation of a large dysmorphic nucleus (white arrows) within a CoM (AD case #48). Scale bars: 5 µm.

Supplementary Fig.3 Serial sections of PaM, CoM, CoM-Rod, and clumped microglia. High-resolution confocal acquisitions of immunostainings against microglia (Iba1, magenta) and nuclei (DRAQ7^{TM}, white). Four types of microglia accumulations, shown in 3D (left) with corresponding 2D z-stack images (right). Examples of CoM-Rod, PAM from case #35 (AD), and clumped microglia from case #21 (DLB) and CoM from case #23 (AD). All scale bars: 50 µm.

Supplementary Fig.4 CoM, Tau pathology and surrounding astrocytes. (A) Low and high magnification on a CoM (Iba1, magenta), which is not engulfing any AT8+ structure (AT8, green; DRAQ7^{TM}, cyan) in the hippocampus of an AD case #52. (B) A CoM (Iba1, magenta) is enveloping a Thiazine Red (TR, cyan) positive structure within the CA1 PL of an AD patient sample (case #34). The CoM is spatially distant from reactive astrocytes (GFAP, green). Right panel shows three confocal planes spaced by 5 µm showing the boundaries between complexity of microglial CoM and surrounding astrocytes (AD case #34). Scale bars: (A, B) 200 µm (low mag), 20 µm (high mag).

Supplementary Fig.5 ErbB4 is expressed by PaM. (A, B) Confocal images (A, 3D; B, 2D confocal plane) showing PaM Iba1+ (green) expressing erbB4 (magenta), surrounded by a polarised astrocyte (GFAP,cyan), in the CA1 hippocampus of an AD case #25. Scale bars: (A, B) 10 µm.

Supplementary Fig.6 Rod-shaped microglia in AD. (A) Iba1 (magenta) immunostaining revealing individual rod-shaped microglia (empty triangles) and 'streaks of aligned rod-shaped microglia (full triangles) in the hippocampus of an AD patient (case #35). Nuclei are stained by DNA dye DRAQ7^{TM}. (B, C) Volcano plots of DEGs in microglia AOIs of Rod vs. CoM and PaM, respectively. Thresholds of Log_2FC = 0.5, P-value = 0.05 and FDR = 0.1 are indicated in the graphs. (D, E) Heatmaps of top 30 DEGs in microglia AOIs of Rod vs. CoM and PaM respectively. Log_2FC values are indicated in brackets for each transcript. Scale bar: 100µm.

Supplementary Fig.7 Complement proteins and PCSK9 in the hippocampus and entorhinal cortex of age-matched controls and AD patients. (A) C1q is expressed in pyramidal neurons of the CA1 in CTL cases (case #14), in neurons and near plaques in the entorhinal cortex of AD patients (case#45). (B) C3 expression is very low in CTL hippocampi and found near plaques (black arrows) in the entorhinal cortex of AD patients (cases #8 and #48). (C) PCSK9 is mainly expressed by neurons in CTL hippocampus and entorhinal cortex (case #7), it is found in the plaque vicinity and associated with Astro-PaM in entorhinal cortex (EC) AD (case #44). Scale bars (A, B, C): 30 µm.

Supplementary Fig.8 Perivascular macrophages, T cells and PaM associated Tau pathology in the hippocampus and entorhinal cortex of age-matched controls and AD patients. (A) 4-plex chromogenic IHC for Iba1 (DAB, brown), CD3 (purple), 4G8 (yellow) and CD8 (teal) in the entorhinal cortices of a CTL and AD samples. T cells were rarely seen in CTL (case#15), but were found in and around blood vessels as well as in AD (case# 40), without reaching the vicinity of plaques. (B) 3-plex chromogenic IHC of CD163 (DAB, brown), Iba1 (purple) and 4G8 (teal) showed a high frequency of CD163+ cells within the PaM in AD entorhinal cortex (case #37). Scale bars (A, B): 30 µm.

Supplementary Table 1 Case information on samples used in the study (IHC fluorescent or IHC chromogenic or Digital Spatial Profiling). Samples and their neuropathological reports were provided by brain banks and assessed by a board-certified pathologist.

Case	Pathological diagnosis	Sex	Age at death (years)	PMD (Hours)	ABC or McKeith score
1	CTL	F	86	5.8	A1B1C1
2		F	80	17.5	A0B1C0
3		F	85	12.0	A1B1C1
4		F	89	23.6	A1B1C0
5		M	89	32.3	A0B0C0
6		M	80	13.0	A1B1C1
7		F	83	35.8	A0B0C0
8		F	95	23.8	A0B0C0
9		M	85	26.8	A0B0C0
10		F	78	16.5	A0B0C0
11		M	93	18.0	A0B1C0
12		M	83	16.8	A0B0C0
13		M	89	32.0	A1B1C1
14		F	55	21.1	A0B0C0
15		M	72	7.5	A0B0C0
16		M	76	19.8	A0B0C0
17		M	68	23.4	A0B1C0
18	DLB	M	64	17.2	Neo-cortical
19		F	83	17.8	Neo-cortical
20		M	82	22.0	Neo-cortical diffuse
21		F	81	8.6	Neo-cortical diffuse
22	AD	F	85	24.8	A2B3C3
23		M	80	15.5	A2B2C2
24		M	82	11.1	A2B2C2
25		M	85	31.1	A2B3C2
26		F	83	25.0	A2B3C2
27		M	91	25.0	A2B3C2
28		M	86	27.3	A2B3C2
29		F	96	26.5	A2B3C2
30		M	95	29.5	A2B3C2
31		M	83	43.3	A1B2C1

32		F	81	23.8	A2B3C2
33		M	77	19.4	A2B3C2
34		M	87	10.8	A2B3C2
35		M	87	21.8	A2B3C2
36		M	93	13.9	A2B3C2
37		M	90	14.6	A2B2C2
38		M	90	26.1	A3B2C3
39		F	74	27.6	A2B2C0
40		F	87	17.0	A1B2C2
41		M	90	32.0	A2B2C3
42		M	78	18.0	A3B3C3
43		F	86	39.0	A3B3C2
44		F	73	41.0	A3B3C3
45		M	65	46.0	A3B3C3
46		M	85	21.0	A3B1C1
47		M	66	30.0	A3B3C2
48		F	84	24.0	A3B3C2
49		F	81	41.0	A3B3C2
50		M	76	22.0	A3B3C3
51		M	68	29.0	A3B3C3
52		M	66	20.0	A3B3C3

AD Alzheimer's disease; CTL age-matched control; DLB Dementia with Lewy Bodies; PMD *post-mortem* delay

Supplementary Table 2 Antibodies used in IHC fluorescent, IHC chromogenic and DSP.

ANTIBODIES	SOURCE	IDENTIFIER	DILUTION IHC fluorescent	DILUTION IHC chromogenic	DILUTION Ventana Discovery Ultra
PRIMARY ANTIBODIES					
Mouse monoclonal anti-β-Amyloid, AA17-24 (4G8)	BioLegend	Cat# 800712, RRID: AB_2734548	1:200	-	1:500
Guinea-pig polyclonal anti-GFAP	Synaptic Systems	Cat# 173004, RRID: AB_10641162	1:500	-	-
Rabbit polyclonal anti-Iba1, C-term	Wako	Cat# 019-19741, RRID: AB_839504	1:500	1:1000	1:1000
Goat polyclonal anti-human Iba1/AIF1 (aa 42-56)	LS-Bio	Cat# LS-B2402-50, RRID: AB_1648188	1:400	-	-
Mouse monoclonal anti-pSyn, PSer129 (11A5)	Prothena Biosciences	N/A	1:500	-	-
Mouse monoclonal anti-pTau, Ser202, Thr205 (AT8)	Thermofisher Scientific	Cat# MN1020, RRID: AB_223647	1:500	-	1:400
Rabbit polyclonal anti-NF-κB p65, phosphoS536	Abcam	Cat# ab86299, RRID: AB_1925243	1:200	-	-

Monoclonal mouse anti-pMLKL PSer358 (6F8)	Aviva Systems	Cat# OASG07777, RRID: AB_2923426	1:200	-	-
Mouse monoclonal anti-CD68 (KP1)	BioLegend	Cat# 916104, RRID: AB 2616797	-	1:200	-
Mouse monoclonal anti-STING (OTI4H1)	Thermo Fisher Scientific	Cat# MA5-26030, RRID: AB_2725437	-	1:500	-
Rabbit monoclonal anti-C1q	Abcam	Cat# ab182451, RRID: AB_2732849	1:200	1:500	-
Rabbit polyclonal anti-C4d	Abcam	Cat# ab36075, RRID: AB_726920	-	-	1:100
Rabbit polyclonal anti-C3	Atlas Antibodies	Cat# HPA003563, RRID: AB_1847117	1:200	-	1:100
Rabbit polyclonal anti-ACADS	Sigma Aldrich	Cat# HPA022271, RRID: AB_1844472	1:500	1:750	-
Rabbit polyclonal anti-erbB4	Sigma Aldrich	Cat# HPA012016, RRID: AB_1846541	1:200	-	-
Rabbit polyclonal anti-ITGA6	Atlas Antibodies	Cat# HPA012696, RRID: AB_1851822	1:200	1:200	-
Rabbit polyclonal anti-pTau (Ser 396)	Thermo Fisher Scientific	Cat# 44-752G, RRID: AB_2533745	-	-	1:1000
Rabbit polyclonal anti-SMURF2	Atlas Antibodies	Cat# HPA071508, RRID: AB_2686406	1:100	-	-
Mouse monoclonal anti-PCSK9 (2F1)	Thermo Fisher Scientific	Cat# MA5-32843, RRID: AB_2802490	-	-	1:1000
Rabbit monoclonal anti-CD3 (2GV6)	Ventana Medical Systems	Cat# 790-4341 (also 05278422001), RRID: AB_2335978	-	-	Ready to use*
Rabbit monoclonal anti-CD8 (SP57)	Ventana Medical Systems	Cat# 790-4460 (also 05937248001), RRID: AB_2335985	-	-	Ready to use*
Rabbit monoclonal anti-GFAP (EP672Y)	Ventana Medical Systems	Cat# 760-4345 (also 05269784001), RRID: N/A	-	-	Ready to use*
Mouse monoclonal anti-CD163 (MRQ-26)	Ventana Medical Systems	Cat# 760-4437 (also 05973929001), RRID: AB_2335969	-	-	Ready to use*
SECONDARY ANTIBODIES					
Donkey anti-rabbit Alexa Fluor 555	Invitrogen	Cat# A-31572, RRID: AB_162543	1:400	-	-
Donkey anti-mouse Alexa Fluor 488	Jackson ImmunoResearch Labs	Cat# 715-545-150, RRID: AB_2340846	1:400	-	-
Donkey anti-goat Alexa Fluor 488	Jackson ImmunoResearch Labs	Cat# 705-545-147, RRID: AB_2336933	1:400	-	-
Donkey anti-guinea pig Alexa Fluor 488	Jackson ImmunoResearch Labs	Cat# 706-545-148, RRID: AB_2340472	1:400	-	-
Donkey anti-mouse Alexa Fluor 647	Jackson ImmunoResearch Labs	Cat# 715-605-150, RRID: AB_2340862	1:300	-	-
Donkey anti-goat Alexa Fluor 647	Jackson ImmunoResearch Labs	Cat# 705-605-003, RRID: AB_2340436	1:300	-	-
Donkey anti-guinea pig Alexa Fluor 647	Jackson ImmunoResearch Labs	Cat# 706-605-148, RRID: AB_2340476	1:300	-	-
Goat anti-rabbit Atto 647N	Sigma Aldrich	Cat# 40839, RRID: AB_1137669	1:300	-	-
DISCOVERY OmniMap anti-Rb HRP	Roche	Cat# 760-4311, RRID: AB_2811043	-	-	Ready to use*

DISCOVERY OmniMap anti-Ms HRP	Roche	Cat# 760-4310, RRID: AB_2885182	-	-	Ready to use*
DISOCVERY UltraMap anti-Ms Alk Phos	Roche	Cat# 760-4312	-	-	Ready to use*
DISCOVERY CM DAB kit	Roche	Cat# 760-159	-	-	Ready to use*
DISCOVERY Purple Kit	Roche	Cat# 760-229	-	-	Ready to use*
DISCOVERY Teal HRP kit	Roche	Cat# 760-247	-	-	Ready to use*
DISCOVERY Yellow Kit	Roche	Cat# 760-239	-	-	Ready to use*
DSP ANTIBODIES					
Rabbit monoclonal anti-Iba1/AIF1 (E4O4W) Alexa Fluor 594 conjugate**	Cell Signalling Technology	Cat# 48934, RRID: AB_2923427	-	-	-
Mouse monoclonal anti-GFAP (GA-5) DyLight 594 conjugate**	Novus Biologicals	Cat# NBP2-33184DL594, RRID: AB_2923514	-	-	-
Mouse monoclonal anti-Aβ (MOAB-2) Alexa Fluor 532 conjugate **	Novus Biologicals	Cat# NBP2-13075AF532, RRID: AB_2923428	-	-	-

*Vials ready-to-use purchased from Roche Ventana Medical Systems // ** Concentration of antibodies not provided by NanoString Techonologies

Supplementary Table 3 Materials and resources

REAGENT OR RESOURCE			
**Biological samples**			
Human *post-mortem* FFPE hippocampus and prefrontal cortex	GIE-Neuro-CEB biobank	See Table 1	
Human *post-mortem* PFA-fixed hippocampus	Douglas-Bell Brain Bank and GIE-Neuro-CEB biobank	See Table 1	
**Chemicals, peptides, and recombinant proteins**			**Dilution**
DRAQ7™	Cell Signaling	Cat# 7406	1:100
Thiazine Red 0.2µM	Sigma Chemicals, St. Louis, MO, USA		
SYTO™ 13 Green Fluorescent Nucleic Acid Stains	Invitrogen	Cat# S7575	
ProLong™ Gold Antifade Mountant	Invitrogen	Cat# P36930	
**Deposited data**			
DSP proteomics and transcriptomics raw data	This paper	Data available on request	
DSP proteomics and transcriptomics DEP and DEG, ORA	This paper	Supplementary files 7 and 8	
Raw images	This paper	Data available on request	
**Software and algorithms**			
Imaris (Version 9.8.0)	Bitplane	RRID: SCR_007370	
GraphPad Prism (Version 9.4.1)	GraphPad Software, Inc	RRID: SCR_002798	
Biorender	Biorender.com	RRID: SCR_018361	

Huygens STED Deconvolution Software	Scientific Volume Imaging (SVI)	RRID: SCR_014237	
R studio (Version 4.2.1.)	RStudio	RRID: SCR_001905	
QuPath (Version 0.3.2)	qupath.github.io	RRID: SCR_018257	
STRING	http://string.embl.de/	RRID: SCR_005223	
DSP GeoMx Analysis suite (Version 2.5.1.145)	NanoString	RRID: SCR_021660	
**Other**			
Dako Omnis Autostainers	Dako		
IntelliSite Ultra Fast Scanner	Philips		
Ventana Discovery Ultra Autostainer	Roche Diagnostics		

Supplementary File 2- Supplementary Movie 1 CoM serial. Example from an 87-year-old male AD patient (case #35).

Supplementary File 3- Supplementary Movie 2 STED PAM serial. Example from a 66-year-old male AD patient (case #52)

Supplementary File 4- Supplementary Movie 3 CoM pTau serial. Example from an 80-year-old male AD patient (case #23).

Supplementary File 5- Supplementary Movie 4 CoM-TR. Example from an 87-year-old male AD patient (case #34).

Supplementary File 6- Supplementary Movie 5 CoM pSyn serial. Example from an 87-year-old male AD patient (case #35).

Supplementary File 7- Supplementary Excel 1. Spatial profiling DEPs and DEG.

Supplementary File 8- Supplementary Excel 2. ORA.

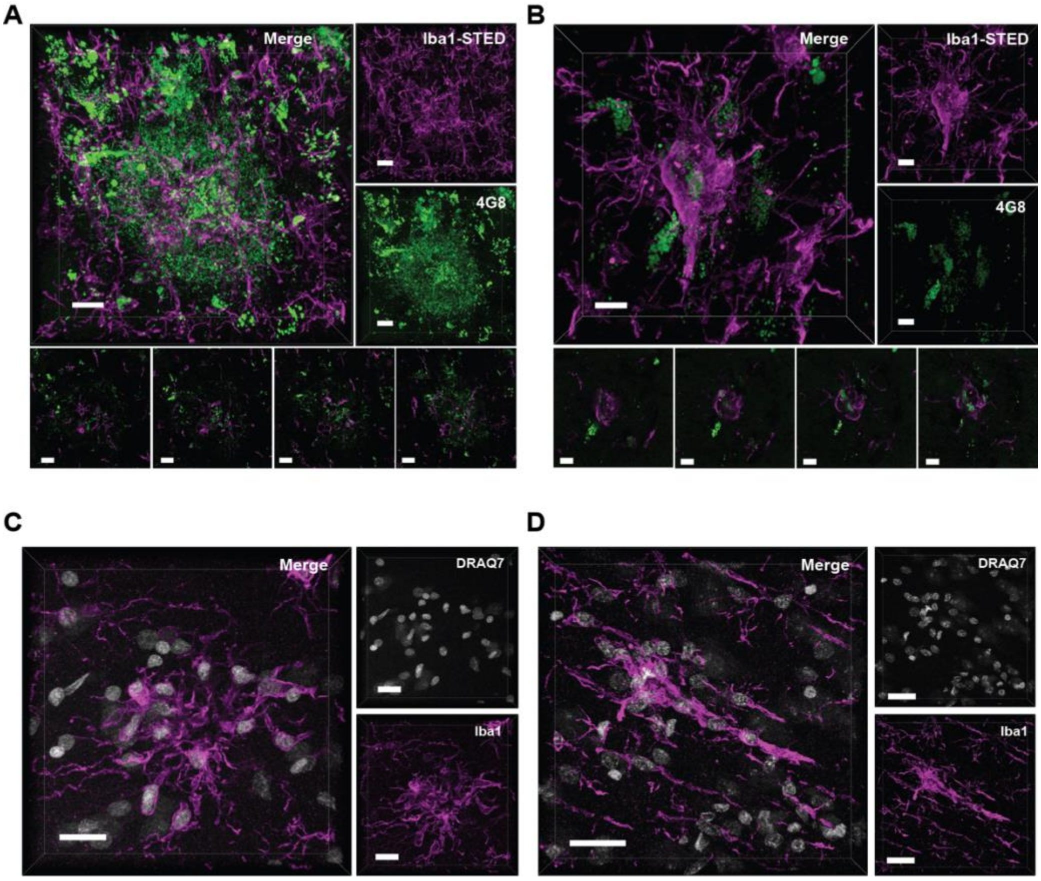

Supplementary Fig.1

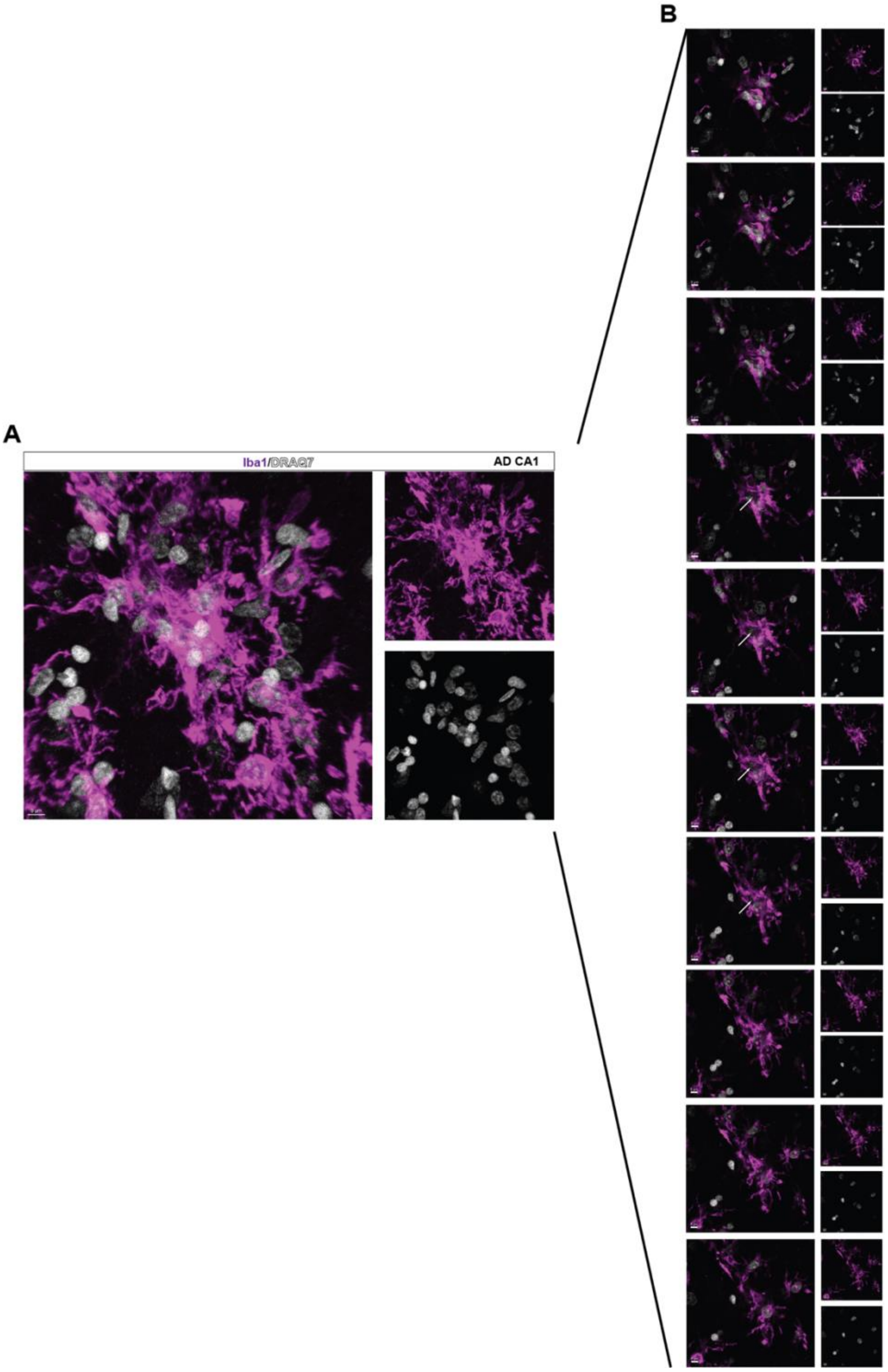

Supplementary Fig.2

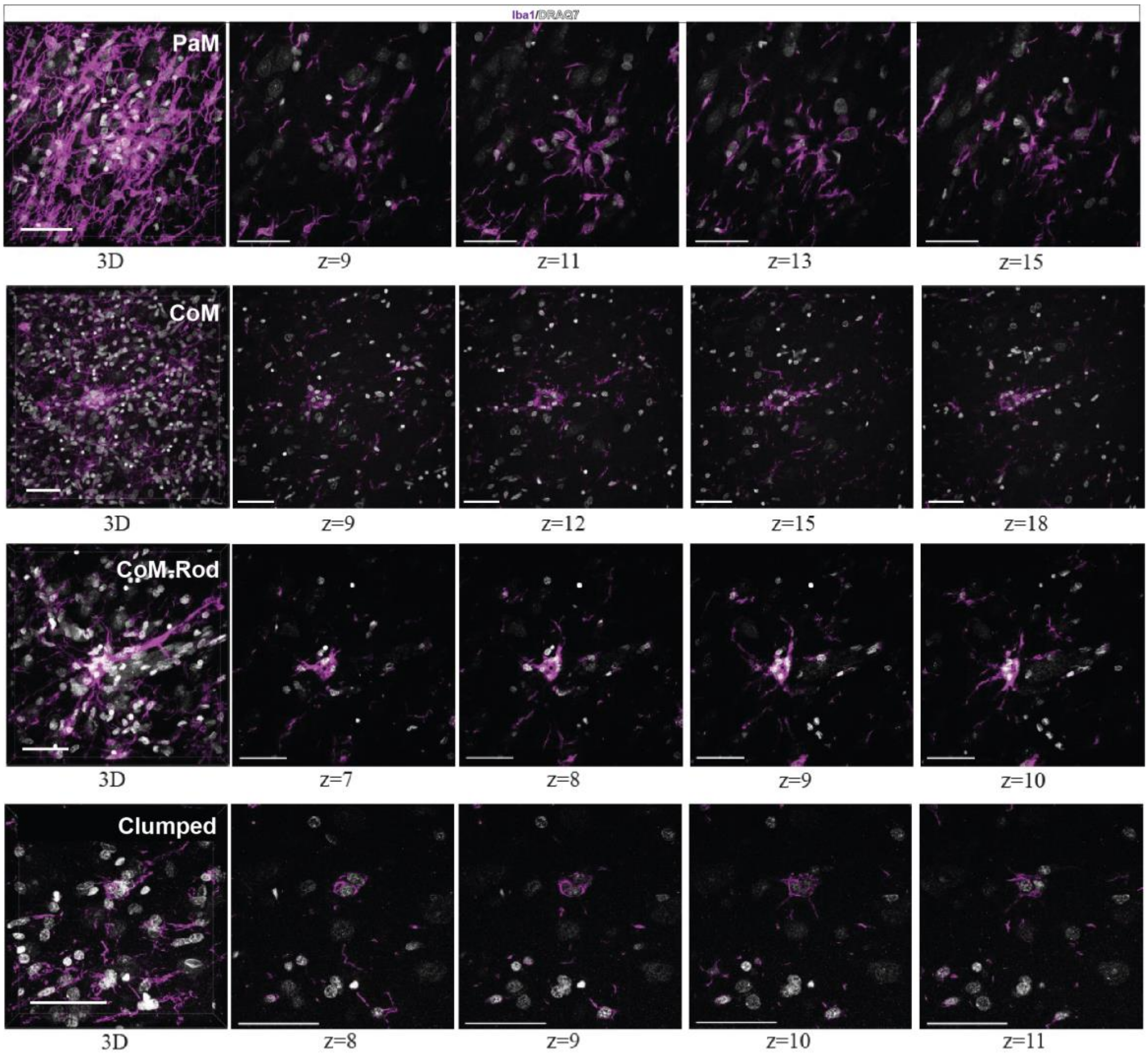

Supplementary Fig.3

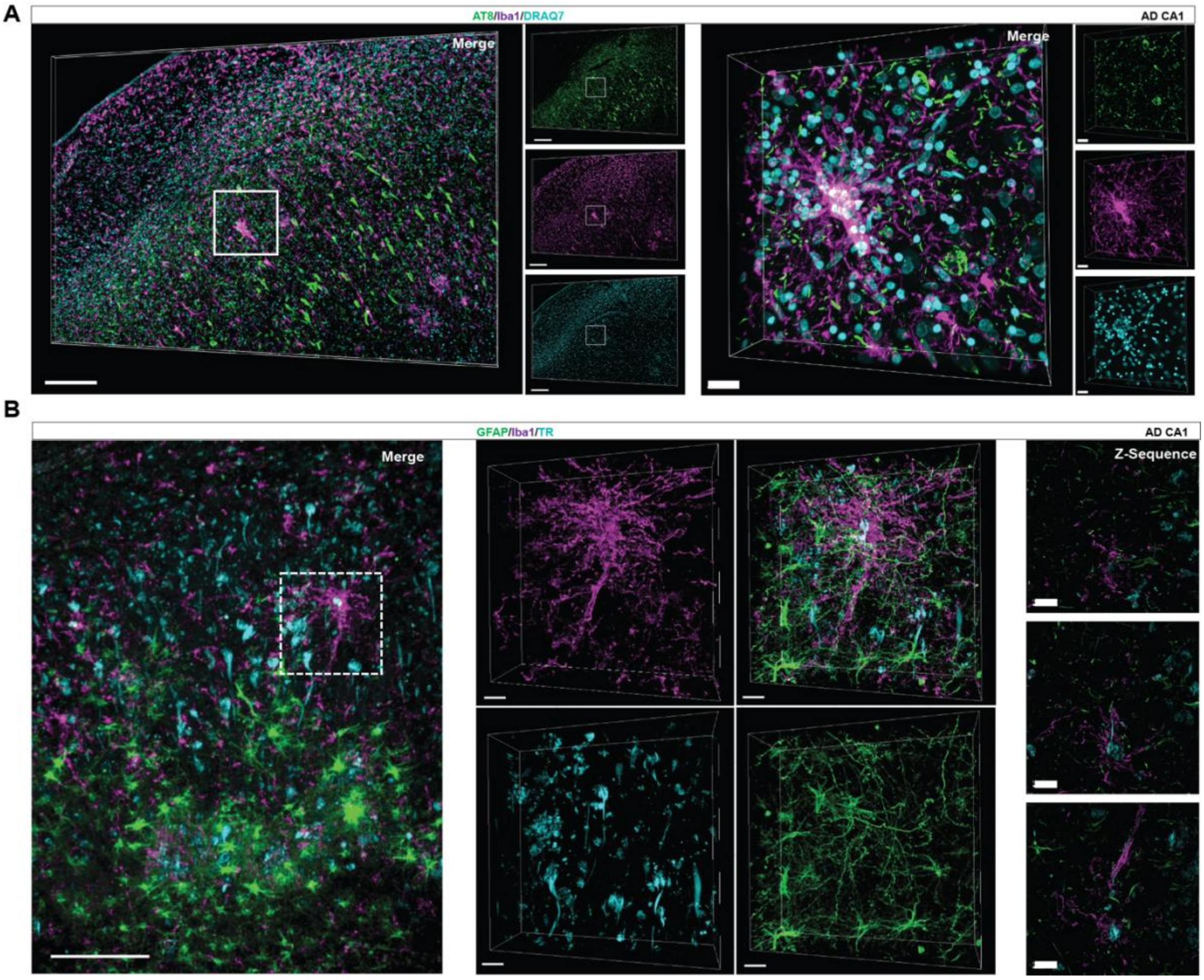

Supplementary Fig.4

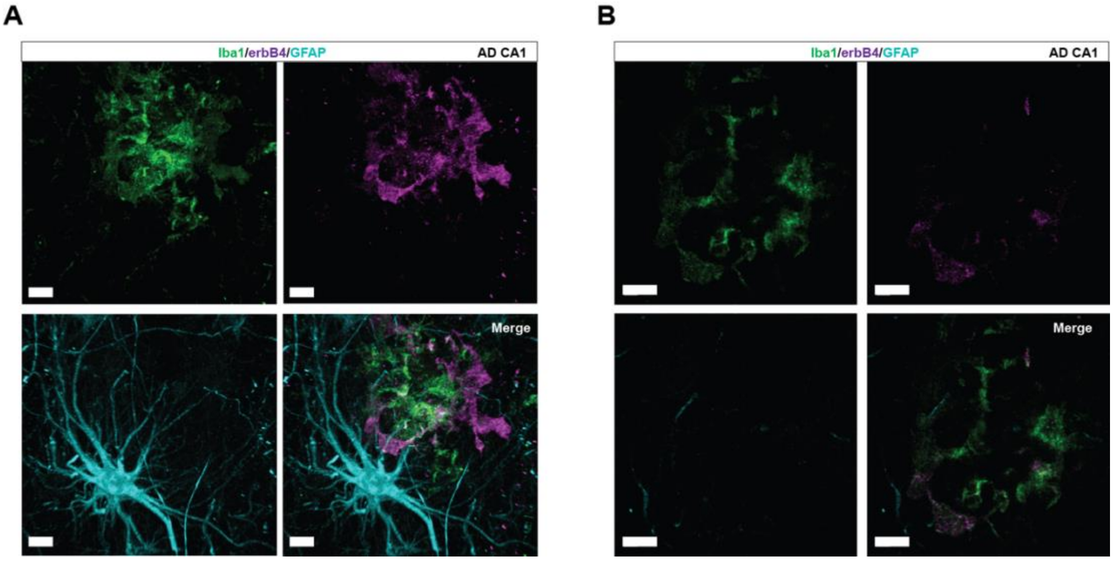

Supplementary Fig.5

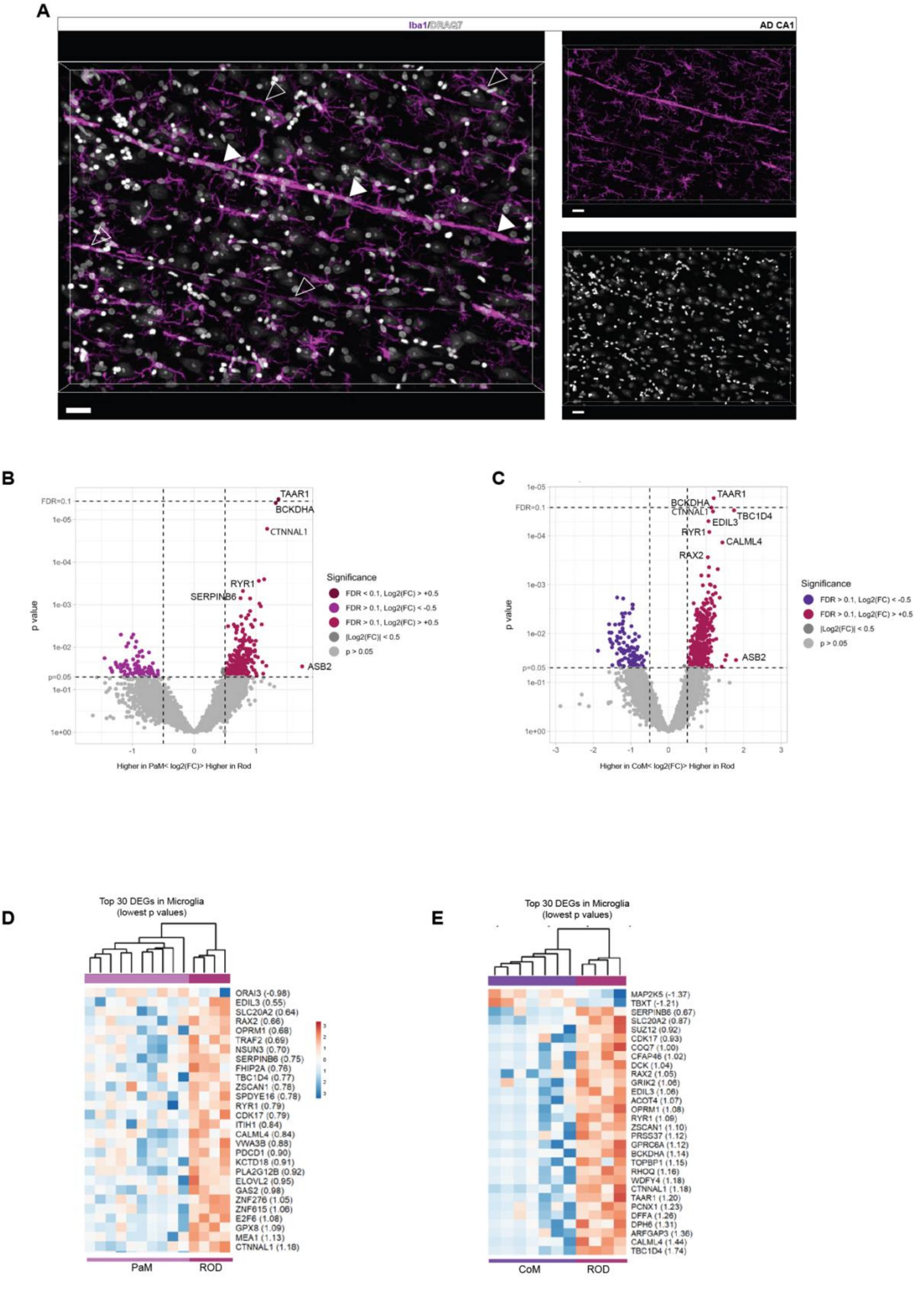

Supplementary Fig.6

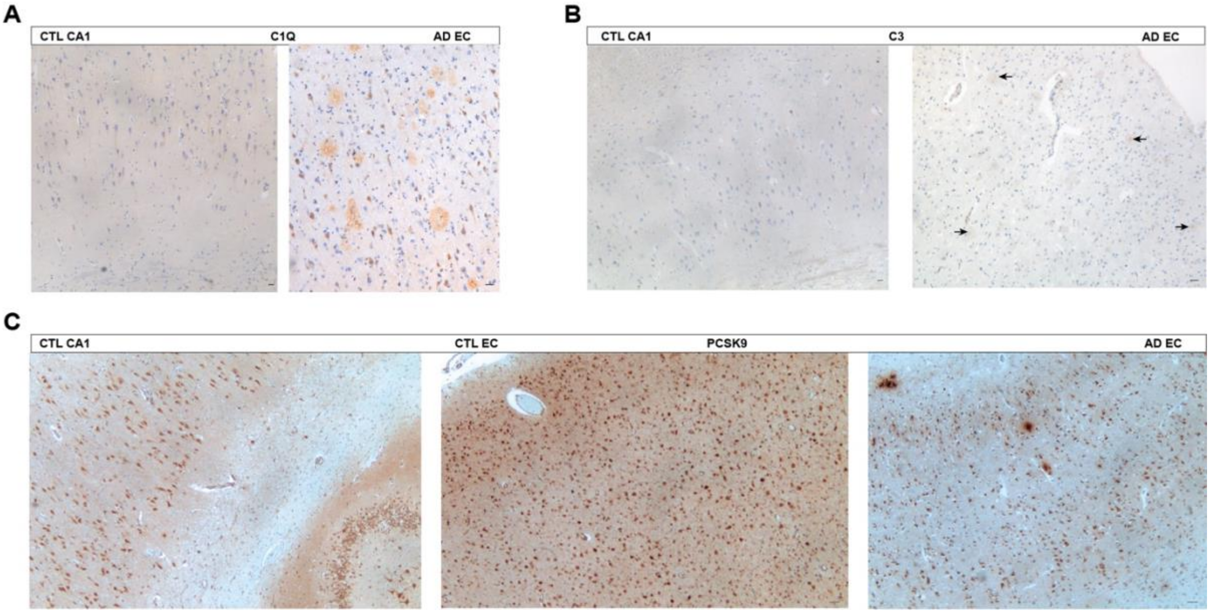

Supplementary Fig.7

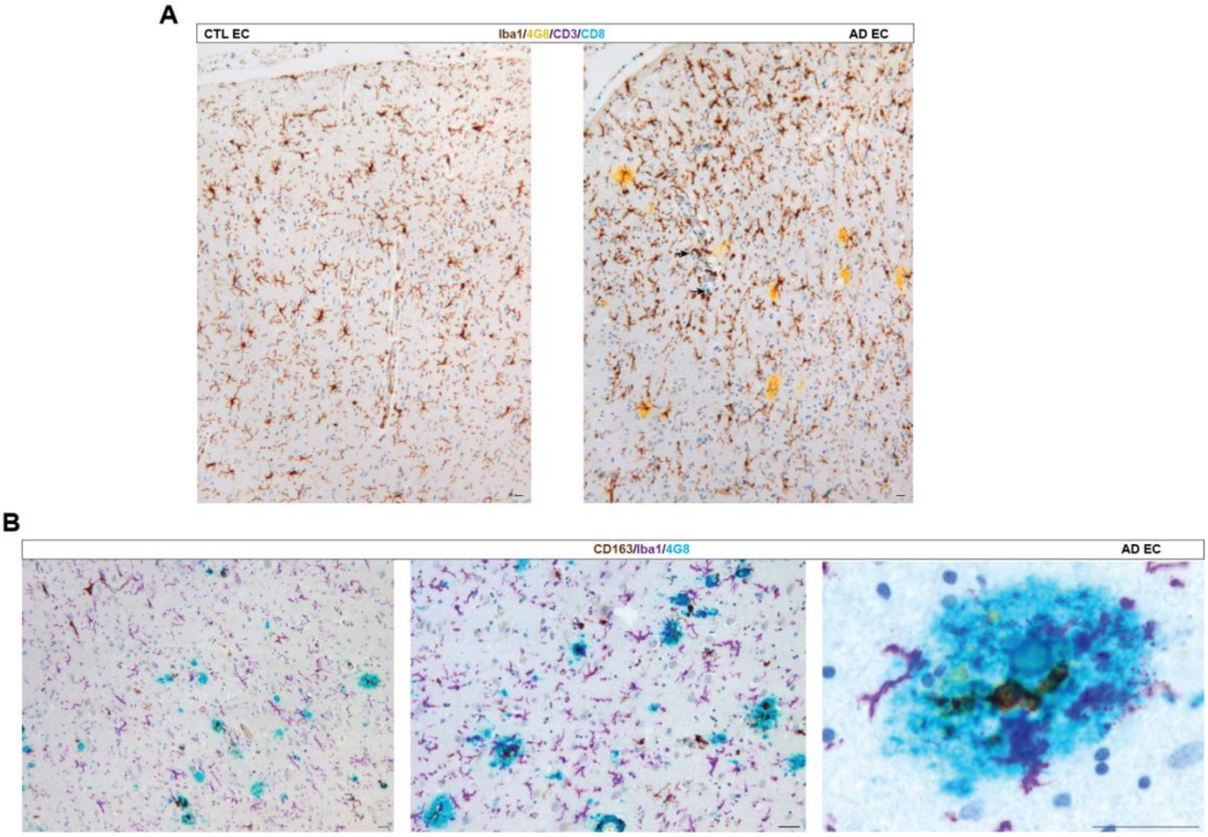

Supplementary Fig.8
